# Two new methods to fit models for network meta-analysis with random inconsistency effects

**DOI:** 10.1186/s12874-016-0184-5

**Published:** 2016-07-28

**Authors:** Martin Law, Dan Jackson, Rebecca Turner, Kirsty Rhodes, Wolfgang Viechtbauer

**Affiliations:** MRC Biostatistics Unit, Cambridge, UK

**Keywords:** Network meta-analysis, Importance sampling, Random inconsistency effects, Informative priors

## Abstract

**Background:**

Meta-analysis is a valuable tool for combining evidence from multiple studies. Network meta-analysis is becoming more widely used as a means to compare multiple treatments in the same analysis. However, a network meta-analysis may exhibit *inconsistency*, whereby the treatment effect estimates do not agree across all trial designs, even after taking between-study heterogeneity into account. We propose two new estimation methods for network meta-analysis models with random inconsistency effects.

**Methods:**

The model we consider is an extension of the conventional random-effects model for meta-analysis to the network meta-analysis setting and allows for potential inconsistency using random inconsistency effects. Our first new estimation method uses a Bayesian framework with empirically-based prior distributions for both the heterogeneity and the inconsistency variances. We fit the model using importance sampling and thereby avoid some of the difficulties that might be associated with using Markov Chain Monte Carlo (MCMC). However, we confirm the accuracy of our importance sampling method by comparing the results to those obtained using MCMC as the gold standard. The second new estimation method we describe uses a likelihood-based approach, implemented in the *metafor* package, which can be used to obtain (restricted) maximum-likelihood estimates of the model parameters and profile likelihood confidence intervals of the variance components.

**Results:**

We illustrate the application of the methods using two contrasting examples. The first uses all-cause mortality as an outcome, and shows little evidence of between-study heterogeneity or inconsistency. The second uses “ear discharge" as an outcome, and exhibits substantial between-study heterogeneity and inconsistency. Both new estimation methods give results similar to those obtained using MCMC.

**Conclusions:**

The extent of heterogeneity and inconsistency should be assessed and reported in any network meta-analysis. Our two new methods can be used to fit models for network meta-analysis with random inconsistency effects. They are easily implemented using the accompanying R code in the Additional file 1. Using these estimation methods, the extent of inconsistency can be assessed and reported.

**Electronic supplementary material:**

The online version of this article (doi:10.1186/s12874-016-0184-5) contains supplementary material, which is available to authorized users.

## Background

Meta-analysis is an established tool for pooling evidence from multiple studies. In conventional meta-analyses, each study contributes a single estimated effect to the analysis. In this conventional univariate setting, the estimated effects of the studies are given weights based on the studies’ precisions, where more precise studies receive more weight. The estimated effects are then pooled by taking a weighted average using these weights, to give a single estimated effect that is intended to describe the population of studies.

The simplest model that is typically used in meta-analysis is the common-effects (or “fixed effect") model. In this model, it is assumed that there is one true treatment effect which is measured to within-study statistical error by every study. However, in many applications, this assumption is implausible as between-study heterogeneity is often observed and/or thought to be present. In such cases, a random-effects model [1] is often employed. In a random-effects model, the true underlying treatment effects from each study are assumed to come from a common distribution whose expectation describes the average effect in the population. We seek to estimate the parameters of this common distribution in order to make statistical inferences.

Network meta-analysis [2, 3] is an extension of conventional meta-analysis that allows the comparison of multiple treatments in a single analysis. Network meta-analysis is particularly useful as multiple treatments can be compared even when there is little or no direct evidence available for some pairs of treatments. A network meta-analysis may be either arm-based or contrast-based and the debate concerning which approach is to be preferred is sometimes fierce [4, 5]. Briefly, arm-based analyses model the average outcome data in each treatment arm of each study; for example, the mean of a continuous measurement or the log odds of success on a treatment arm. See Piepho et al. [6] for a closely-related discussion on this issue. Contrast-based analyses instead model estimated treatment effects using study-specific reference treatments, for example, the mean difference between continuous measurements or the log odds ratio of success in one treatment arm compared to another. In this paper we use a contrast-based approach, which is the more conventional approach in meta-analysis and other settings [4].

As in a standard meta-analysis, it is important to account for between-study heterogeneity in a network meta-analysis and so a random-effects approach is typically adopted (in the absence of heterogeneity, the model collapses to a common-effects model, as we will see in our first example below). An additional issue in the context of a network meta-analysis is the potential for a lack of consistency in the estimated effects: For example, in a single three-arm trial of treatments A, B, and C, it is necessarily true that the relative effect of treatment C compared to A is equal to the estimated relative effect of C compared to B plus the relative effect of B compared to A (i.e., “C – A = (C – B) + (B – A)"), on an appropriate scale. This property is known as “consistency”. In this case, all treatments have been compared directly. A network meta-analysis, which includes multiple studies providing a combination of direct and indirect evidence about the relative effects of various subsets of the treatments of interest, does not necessarily possess this property across the entire network. The absence of consistency in such a network is termed “inconsistency”, and is the result of discrepancies between direct and indirect treatment effect estimates.

A recently proposed model that allows for inconsistency in network meta-analysis is Jackson’s model [7]. This model includes an inconsistency variance component, whose magnitude describes the extent of the inconsistency across the network. In this model, inconsistency is treated as an additional source of variation alongside between-study heterogeneity, and is conceptualised and estimated in a similar manner. To date, two estimation methods have been proposed to fit this model: The first of these methods [8] uses a Bayesian framework and Markov Chain Monte Carlo (MCMC) methods as implemented in Win/OpenBUGS [9]. The second of these methods [7] is an extension of the method for conventional random-effects meta-analysis proposed by DerSimonian and Laird [1] and is based on the decomposition of the Q statistic by Krahn et al. [10].

The primary contribution of the present paper is to introduce two new estimation methods for fitting Jackson’s model. The first of these new methods implements the type of Bayesian meta-analysis suggested by Jackson et al. [8] but here we propose using importance sampling, rather than MCMC, to sample from the posterior distribution and further, we make use of informative prior distributions for the unknown variance components. We consider this new method an improvement over the previous Win/OpenBUGS implementation for two reasons. First, by using importance sampling techniques, we avoid difficulties associated with ‘burning-in’ the chains and autocorrelation; we still encounter Monte Carlo error however. For those who prefer to use MCMC, importance sampling provides reassurance that the MCMC estimates are reliable. Second, we take a fully Bayesian approach, incorporating external information on unknown variance components, where we are able to justify and defend our choice of priors. Unknown variance components are difficult to estimate and using truly vague prior distributions makes these, at best, extremely difficult to identify [11]. By using appropriate informative priors, we are able to overcome some of these theoretical and practical difficulties that are associated with Bayesian analyses that attempt to use vague priors, though these benefits come at the cost of additional assumptions via the prior distributions.

The second new estimation method we describe uses a likelihood-based approach to fit the model. We propose this as an improvement to the previous classical estimation method that is based on the method of moments [7]. Although the method of moments retains its advantages of computational simplicity and semi-parametric nature, likelihood-based methods are based on sufficient statistics and so make use of all the available information in the sample. In addition, inferences for variance components are easily obtained in a likelihood framework.

The rest of the paper is set out as follows. We start by describing our model for network meta-analysis and explain how this may be modified to incorporate alternative models with random inconsistency effects. We then describe our two new estimation methods. We apply these estimation methods to two contrasting examples. We then conclude with a discussion.

## Methods

In this section we describe the model and the two new corresponding estimation methods. We will also explain how to modify the methods to fit alternative models for network meta-analysis with random inconsistency effects. We use A, B, C, etc. to denote the treatment groups and *d* to denote the study ‘design’, where the term ‘design’ refers to the sets of treatment comparisons included in the study; for example, all studies that compare the treatments A, B, and C (and no others) belong to the same design, and if this is the first design then *d*=1 is taken to mean the ‘ABC design’. Our primary inferential interest is making inferences about the average treatment differences (i.e., A vs. B, A vs. C, B vs. C, and so on) among all the treatments included in the network.

### The model

Jackson’s model has been fully described previously [7], though we present it briefly here for completeness. This model is a special case of the model proposed by Jackson et al. [8], which in turn is a special case of the design-by-treatment interaction model [12, 13]. Briefly, the design-by-treatment interaction model was inspired by loop-inconsistency models [14] which add inconsistency parameters to closed loops where inconsistencies may arise; loop inconsistency models therefore directly appeal to intuitive notions of inconsistency in network meta-analysis. However, the form of the loop inconsistency model, and therefore the inconsistencies that are described, depends on the treatment ordering [12, 15]. In order to overcome this issue, the design-by-treatment interaction model was developed [12, 13], which allows every design to estimate a different set of treatment effects. The inclusion of a design-by-treatment interaction term has also been explored by Piepho [16].

Jackson et al. [8] suggested making particular parametric assumptions for the between-study heterogeneity and inconsistency variance components and Jackson et al. [7] further simplified matters by using normal within-study approximations where the within-study covariance matrices are treated as fixed and known. It is the modelling framework of Jackson et al. [7] that we take as our basis here and this model is given by

(1) $$  \mathbf{Y}_{{di}} = \boldsymbol{\delta}_{d} + \mathbf{B}_{{di}} + \boldsymbol{\omega}_{d} + \boldsymbol{\epsilon}_{{di}},  $$

where **Y**_*di*_ represents the *c*_*d*_×1 vector of estimated treatment effects from study *i* of design *d*, where *c*_*d*_ is the number of treatment arms in design *d* minus one. To conveniently define the estimated treatment effects in **Y**_*di*_, we choose a design-specific baseline treatment group (e.g., A in the ABC design, B in the BC design). The entries of **Y**_*di*_ are then obtained as the estimated treatment effects of the *c*_*d*_ treatment groups, in alphabetical order, compared to the baseline treatment group (e.g., A vs. B and A vs. C in the ABC design). Without loss of generality, we take the design-specific baseline treatment group as the treatment that appears first in the alphabet in each design, and we assume that this choice has been made throughout, though any reference treatment may be chosen for each design. Model (1) has four components, and we describe these next. See also previous accounts of this modelling framework [7, 8] for further details.

We choose a reference treatment A and we denote the average (i.e., across all designs and studies) treatment effects in the network meta-analysis as *δ*^*AB*^, *δ*^*AC*^, and so on, where we refer to these parameters as ‘basic parameters’. Then ***δ***_*d*_ represents the average treatment effects of design *d* in terms of the basic parameters; for example, for the design that includes treatments C, D and E only, ***δ***_*d*_=(*δ*^*AD*^−*δ*^*AC*^,*δ*^*AE*^−*δ*^*AC*^)^*T*^. We make inferences about treatment effects that are not relative to the reference treatment A by making inferences about appropriate contrasts of the basic parameters, so that our framework allows us to make inferences about any pair of treatments included in the network.

### The variance components

The between-study heterogeneity is modelled by the random effects vector **B**_*di*_, where we assume that $\mathbf {B}_{{di}} \sim N(\mathbf {0}, \tau ^{2}_{\beta } \mathbf {P}_{c_{d}})$, where $\mathbf {P}_{c_{d}}$ is a square matrix of dimension *c*_*d*_ with ones on the diagonal elements and halves everywhere else. Similarly, the inconsistency is modelled by ***ω***_*d*_, where $\boldsymbol {\omega }_{d} \sim N(\mathbf {0}, \tau ^{2}_{\omega } \mathbf {P}_{c_{d}})$. The ***ω***_*d*_ are called the inconsistency effects and we make a distributional assumption for these parameters.

The distributional assumptions made for the between-study heterogeneity (i.e., **B**_*di*_) and the inconsistency random effects (i.e., ***ω***_*d*_) are motivated primarily because they lead to a particularly simple model form and because it is difficult to identify more complex models in the majority of applications [7, 8]. It is the ***ω***_*d*_ that allow the different designs to estimate different sets of treatment effects, so that the ***ω***_*d*_ provide the design-by-treatment interaction. The two unknown variance components, $\tau ^{2}_{\beta }$ and $\tau ^{2}_{\omega }$, describe the extent of the additional (to the within-study) variation in the data that is due to between-study variation and inconsistency, respectively. If $\tau ^{2}_{\omega }=0$, then, to within-study and conventional between-study variation, all studies of all designs estimate the same set of treatment effects (because then all ***ω***_*d*_ = ***0***). Hence $\tau ^{2}_{\omega }=0$ is taken to indicate consistency. If instead $\tau ^{2}_{\omega }>0$, then the designs estimate different treatment effects and the network is inconsistent, and then the treatment effects in the ABC loop would not ‘add up’ in the way implied by the consistency assumption. We refer to $\tau ^{2}_{\beta }$ and $\tau ^{2}_{\omega }$ as the between-study variance and the inconsistency variance, respectively.

The within-study variation is modelled by ***ε***_*di*_, where we assume that ***ε***_*di*_∼*N*(**0**,**S**_*di*_), where **S**_*di*_ is the within-study covariance matrix. The within-study covariance matrix is estimated in practice but is treated as fixed and known in the analysis. If all studies are two-arm studies, and so estimate a single treatment effect, model (1) is equivalent to Lumley’s model [17].

### The full model

Model (1) describes the estimated effects for each study. In order to describe the entire dataset, we vertically stack the **Y**_*di*_ to create **Y**, where model (1) implies that

(2) $$  \mathbf{Y} \sim N\left(\mathbf{X} \boldsymbol{\delta}, \, \tau^{2}_{\beta} \mathbf{M}_{1} + \tau^{2}_{\omega} \mathbf{M}_{2} + \mathbf{S}\right),  $$

where ***δ*** is a vector that contains the basic parameters. This vector is premultiplied by design matrix **X**, where the design matrix ensures that model (2) provides the mean structure implied by model (1). The block diagonal matrix **S** contains the **S**_*di*_ along the main block diagonal, so that the within-study distributions in models (1) and (2) are equivalent. Similarly, the terms $\tau ^{2}_{\beta } \mathbf {M}_{1}$ and $\tau ^{2}_{\omega } \mathbf {M}_{2}$ ensure the equivalence between the between-study and inconsistency variance structures, respectively, in models (1) and (2). This is achieved by defining square matrices **M**_1_ and **M**_2_: **M**_1_ contains ones on the main diagonal, so that **M**_1*ii*_=1 for all *i*. Furthermore, for *i*≠*j*, **M**_1*ij*_=1/2 if the corresponding entries (i.e., rows) of **Y** are from the same study, and **M**_1*ij*_=0 otherwise. We similarly define **M**_2_ as containing ones on the main diagonal, so that **M**_2*ii*_=1 for all *i*. Furthermore, for *i*≠*j*, **M**_2*ij*_=1 if the corresponding entries of **Y** are from the same design and refer to the same treatment effect, **M**_2*ij*_=1/2 if they are from the same design but refer to different treatment effects, and **M**_2*ij*_=0 otherwise. In the previous presentation of the model [7] we used **P**_1_ and **P**_2_ to denote **M**_1_ and **M**_2_, but we now use **M** to denote these matrices, in order to avoid a clash of notation with $\mathbf {P}_{c_{d}}$. Matrices **M**_1_ and **M**_2_ for example two are given in the Additional file 2 for illustration.

Our methodology can be used to fit other models for network meta-analysis with random inconsistency effects by modifying matrix **M**_2_. We could also make alternative assumptions about the form of the between-study heterogeneity by modifying **M**_1_. Furthermore, we could incorporate further unknown variance parameters, by introducing more products of **M** matrices and these unknown variances. In particular, this could allow models that use more than one parameter to describe the between-study heterogeneity. However, introducing more than two unknown variance components will make the model harder to identify and interpret.

### The standard method for making inferences about the average treatment effects in a classical framework

The standard method for making inferences about the average treatment effects (across all studies of all designs) in a classical framework initially estimates the unknown variance components and then treats them as known in the analysis [7, 18]. The analysis then proceeds as a weighted regression model, where all weights are regarded as known. We denote classical estimates of the two unknown variances as $\widehat {\tau }^{2}_{\beta }$ and $\widehat {\tau }^{2}_{\omega }$. We then define the estimated total variance of **Y** to be $\mathbf {V} = \widehat {\tau }^{2}_{\beta } \mathbf {M}_{1} + \widehat {\tau }^{2}_{\omega } \mathbf {M}_{2} + \mathbf {S}$. The vector of basic parameters is then estimated as

(3) $$  \boldsymbol{\hat{\delta}} = (\mathbf{X}^{T} \mathbf{V}^{-1} \mathbf{X})^{-1} \mathbf{X}^{T} \mathbf{V}^{-1} \mathbf{Y},  $$

which is approximately normally distributed, centred at ***δ***, with covariance matrix

(4) $$  \text{Var}(\boldsymbol{\hat{\delta}}) = (\mathbf{X}^{T} \mathbf{V}^{-1} \mathbf{X})^{-1}.  $$

Hence statistical inference for the basic parameters, such as hypothesis testing and calculating confidence intervals, is easily performed. Inferences for average treatment effects that compare pairs of treatments that do not include treatment A can be obtained by applying this normal approximation to appropriate contrasts of the basic parameters.

This conventional method does not take into account the uncertainty in the unknown variance components, which can have unfortunate implications for the accuracy of the statistical inference in small samples [7, 18]. Provided that this concern is not too serious however, the main statistical difficulty lies in estimating ${\tau }^{2}_{\beta }$ and ${\tau }^{2}_{\omega }$. The method of moments was previously proposed for this purpose [7] but we show how to use likelihood-based methods, and in particular restricted maximum likelihood (REML), for this purpose below.

### Bayesian methods

Bayesian analysis differs from frequentist analysis by treating unknown parameters as random quantities and assigning prior distributions to those quantities. These prior distributions are combined with the data to make inferences on the unknown parameters. It is necessary to choose prior distributions for all parameters in a Bayesian analysis. We will use informative independent lognormal prior distributions for $\tau ^{2}_{\beta }$ and $\tau ^{2}_{\omega }$, which we denote as $\tau ^{2}_{\beta } \sim LN\left (\mu _{\beta }, \sigma _{\beta }^{2}\right)$ and $\tau ^{2}_{\omega } \sim LN\left (\mu _{\omega }, \sigma _{\omega }^{2}\right)$. The choice of hyperparameters $\left (\text {i.e}., \mu _{\beta }, \sigma _{\beta }^{2}, \mu _{\omega }, \sigma _{\omega }^{2}\right)$ is discussed below. Informative priors were chosen for the unknown variance components because these are hard to identify in typical meta-analysis datasets with small numbers of studies. We follow Turner et al. [19] in using lognormal priors because they provide concrete proposals for priors of this type. We use independent uniform priors for the basic parameters ***δ***, specified over a wide range to include the parameter space where the likelihood is non-negligible. These uniform priors are intended to be vague, which reflects our lack of prior knowledge regarding the parameter estimates, and choosing uniform rather than diffuse normal priors simplifies the resulting posterior distributions.

### Informative priors for heterogeneity

We directly follow Turner et al. [19] in using informative lognormal priors for the between-study variance, $\tau ^{2}_{\beta }$. Briefly, Turner et al. used the Cochrane Database of Systematic Reviews to model binary outcome data in 14,886 meta-analyses, and subsequently obtained predictive distributions for the between-study heterogeneity variance. Their proposal is to use these predictive distributions as priors in random-effects meta-analyses [20]. Their predictive distributions cover eighty distinct settings, comprising a combination of sixteen outcome types (e.g., all-cause mortality, adverse events) and five types of intervention comparisons (e.g., pharmacological vs. placebo/control). We have no reason to believe that the extent of heterogeneity in pairwise meta-analyses would be different if these trials were part of a network. Thus, in the absence of informative priors for network meta-analysis we consider it sensible to use the distributions of Turner et al. [19].

Based on the settings of our example datasets (see below), the appropriate context-specific lognormal prior distributions were chosen for the heterogeneity variance [19]. For the first example dataset, the lognormal prior $\tau ^{2}_{\beta } \sim LN(-3.50, 1.26^{2})$ (2.5 %, 50 %, 97.5 % quantiles (0.00, 0.03, 0.36)) was chosen, reflecting the outcome of all-cause mortality and the comparison of non-pharmacological treatments. For the second example dataset, the lognormal prior $\tau ^{2}_{\beta } \sim LN(-2.29, 1.58^{2})$ (2.5 %, 50 %, 97.5 % quantiles (0.00, 0.10, 2.24)) was chosen, again reflecting the type of outcome, “symptoms reflecting continuation of condition", and pharmacological interventions.

### Informative priors for inconsistency

Turner et al. [19] did not fit their model to network meta-analysis datasets and so did not include inconsistency as a source of additional variation. As a consequence, their priors are not immediately applicable to the other unknown variance component in our model, $\tau _{\omega }^{2}$. In order to make use of their informative priors, we suggest a simple and intuitive way to translate the priors of Turner et al. [19] for $\tau _{\beta }^{2}$ into priors for $\tau _{\omega }^{2}$. In what follows, we make frequent use of the standard results that a lognormal distribution, *LN*(*μ*,*σ*^2^), has a mean of exp(*μ*+*σ*^2^/2) and a variance of (exp(*σ*^2^)−1) exp(2*μ*+*σ*^2^). We use a lognormal prior for $\tau ^{2}_{\omega }$, because it seems reasonable to assume that inconsistency follows a similarly-shaped distribution to heterogeneity.

Our method for translating priors for $\tau _{\beta }^{2}$ into priors for $\tau _{\omega }^{2}$ requires the analyst to choose a factor by which to scale the mean of the prior for $\tau ^{2}_{\beta }$, in order to obtain the mean of the prior for $\tau ^{2}_{\omega }$. From the standard results above for the lognormal distribution, we can easily obtain the mean and variance of the prior distribution of $\tau ^{2}_{\beta }$. We set the variance of the prior distribution for $\tau ^{2}_{\omega }$ equal to that of $\tau ^{2}_{\beta }$, to reflect a similar degree of uncertainty in the two unknown variances; we denote this variance as *V*. We then obtain the mean *M* of the prior distribution of $\tau ^{2}_{\omega }$ as the product of the scale factor and the mean of the prior distribution of $\tau ^{2}_{\beta }$. Then solving simultaneous equations using the standard results for the lognormal distribution, we obtain the required hyperparameters that provide mean *M* and variance *V* for the prior distribution of $\tau ^{2}_{\omega }$ as

(5) $$  {\small{\begin{aligned} {}\mu_{\omega} = \log(M) - \frac{1}{2}\left[ \log\left(\frac{V}{M^{2}} + 1 \right) \right],\quad \sigma_{\omega}^{2} = \log\left(\frac{V}{M^{2}} + 1\right). \end{aligned}}}  $$

As an example, the lognormal prior for heterogeneity in the first example is *LN*(−3.50,1.26^2^). This prior distribution has a mean of exp(−3.50+1.26^2^/2)=0.067 and variance of (exp(1.26^2^)−1) exp(2×−3.50+1.26^2^)=0.017 (both rounded). Suppose we then decide to assume that the inconsistency variance $\tau ^{2}_{\omega }$ is likely to be non-negligible, but also that it is likely to be considerably less than the between-study $\tau ^{2}_{\beta }$, and we reflect this by applying a scaling factor of 0.5 to the mean of the prior distribution of $\tau ^{2}_{\beta }$ to obtain an informative prior for $\tau ^{2}_{\omega }$. Then we use *M*=0.067/2=0.0335 and variance *V*=0.017, and (5) gives *μ*_*ω*_=−4.79 and $\sigma _{\omega }^{2}=1.67^{2}$ (both rounded), so that the prior distribution of $\tau ^{2}_{\omega }$ is taken to be *LN*(−4.79,1.67^2^).

We recognise that this way of deriving informative priors for $\tau ^{2}_{\omega }$ is somewhat speculative and, in particular, assumes that the amount of prior information that we have about between-study heterogeneity is similar to the amount of prior information that we have about the inconsistency variance. Since we conceptualise inconsistency as a particular form of additional variation between studies, similarly to the way we conceptualise between-study variation, it is not unreasonable to take the amount of prior knowledge about the likely extent of inconsistency as being similar to the likely extent of the between-study heterogeneity.

Other methods may be employed to characterise the prior for inconsistency. For example, one may wish to specify a lognormal distribution based on the choice of quantiles, rather than choosing a mean and variance. Further, a truncated distribution may be used, to limit the distribution to what is deemed a plausible interval. However, we choose to specify the distribution in terms of its mean and variance, as this makes clear the differences between the chosen priors for heterogeneity and inconsistency, and allows the parameters *μ*_*ω*_ and $\sigma ^{2}_{\omega }$ to be easily found using (5).

In order to present a range of possibilities for our examples below, we perform analyses assuming that the prior mean of the inconsistency variance is equal to that of the between-study heterogeneity variance, assuming that the prior mean of the inconsistency variance is half that of the between-study heterogeneity variance, and finally assuming that the prior mean of the inconsistency variance is one tenth that of the between-study heterogeneity variance. These analyses cover a wide range of possibilities: the first of these analyses is intended to reflect the notion that the inconsistency is as severe as the between-study heterogeneity, whilst the final analysis assumes that the consistency assumption is a reasonable description of the data while still acknowledging the possibility that some inconsistency may be present.

As with the decision to use lognormal prior distributions characterised by mean and variance, other choices may be made with regards to the form of the informative prior for the inconsistency variance. In the main paper we present results using an informative prior with a mean of half of that of the informative prior for heterogeneity. In the Additional file 3, it can be seen for example two that the greater the mean of the prior for inconsistency, the more the point estimate for inconsistency resembles the frequentist estimate in Table 2. However, the intervals of uncertainty around the inconsistency estimates make definitive statements difficult when comparing these variance components. It is possible to use a prior for inconsistency with a greater mean than that of the prior for heterogeneity, however, in the face of high inconsistency we would discourage the use of network meta-analysis.

### Importance sampling

Importance sampling [21] is a technique that can be used for estimation when the posterior distribution is difficult or impossible to obtain analytically, which is the case here. Importance sampling has been used previously to undertake meta-analyses [19, 22]. MCMC is also often used for this purpose and is more powerful than importance sampling. However, importance sampling is adequate for relatively simple models such as ours, and avoids problems associated with burn-in and autocorrelation of the simulated realisations of the posterior distribution. In importance sampling, simulated samples of all parameters are drawn independently from a proxy distribution that is straightforward to sample from, and that ideally resembles the true distribution of interest. However, importance sampling algorithms can fail to converge if the tails of the proxy distribution are too thin. Hence we include a scaling factor *s* in the importance sampling algorithm below to ensure that the proxy distributions have heavy tails. The samples drawn from the proxy distribution are then weighted using the ratio of the density of the true distribution of interest to the proxy density, where these densities are evaluated using the simulated values. Any numerical summary of the simulated values from the proxy distribution, when weighted in this way, give the corresponding summary from the true distribution. In the context of Bayesian statistics, this true distribution that we wish to sample from is the posterior distribution.

In order to describe the importance sampling algorithm that we use, we define some further quantities. We define $\widetilde {\tau }^{2}_{\beta }$ and $\widetilde {\tau }^{2}_{\omega }$ to be the means of the corresponding prior distributions, so that $\widetilde {\tau }^{2}_{\beta } = \text {exp}(\mu _{\beta } + \sigma ^{2}_{\beta }/2)$ and $\widetilde {\tau }^{2}_{\omega } = \text {exp}(\mu _{\omega } + \sigma ^{2}_{\omega }/2)$. We then define $\widetilde {\mathbf {V}} = \widetilde {\tau }^{2}_{\beta } \mathbf {M}_{1} + \widetilde {\tau }^{2}_{\omega } \mathbf {M}_{2} + \mathbf {S}$ which, if we take the estimates of the unknown variance components as their prior means, is the estimated covariance matrix of **Y** from model (2). We then define $\boldsymbol {\widetilde {\delta }} = (\mathbf {X}^{T} \mathbf {\widetilde {V}}^{-1} \mathbf {X})^{-1} \mathbf {X}^{T} \mathbf {\widetilde {V}}^{-1} \mathbf {Y}$ and $\boldsymbol {\widetilde {\delta }}_{{var}} = (\mathbf {X}^{T} \mathbf {\widetilde {V}}^{-1} \mathbf {X})^{-1}$, where $\boldsymbol {\widetilde {\delta }}$ and $\boldsymbol {\widetilde {\delta }}_{{var}}$ are the corresponding classically estimated vector of basic parameters and its covariance matrix, respectively. We condition on the observed data in a Bayesian analysis, which means that the above quantities denoted using the ‘tilde’ notation are treated as constants.

We propose simulating from the following multivariate proxy distribution in our importance sampling algorithm, where we simulate each of these three random variables independently:

(6) $$  {\small{\begin{aligned} {}\boldsymbol{\delta}^{*} \!\sim\! N \left(\boldsymbol{\widetilde{\delta}}, \: s \,\boldsymbol{\widetilde{\delta}}_{{var}} \right), \; \;\tau^{2*}_{\beta} \sim LN\left(\mu_{\beta}, \sigma_{\beta}^{2}\right), \;\; \tau^{2*}_{\omega} \sim LN\left(\mu_{\omega}, \sigma_{\omega}^{2}\right), \end{aligned}}}  $$

where *s* denotes the scaling factor used in the importance sampling algorithm. The asterisks in Eq. (6) indicate a simulated random draw from the proxy distribution. The intuition that underlies the proxy distribution in (6) is that samples in meta-analysis are often small and so contain little information about the unknown variance components. Hence the priors of $\tau ^{2}_{\beta }$ and $\tau ^{2}_{\omega }$ are likely to resemble their posteriors, but the priors can be expected to have heavier tails, as desired in importance sampling algorithms, because the posteriors include both the information in the prior and the data. Hence the priors of the unknown variance components are highly suitable as proxy distributions and their use simplifies the formula for the weights below.

The proxy distribution of ***δ***^∗^ is motivated by the standard method for making inferences in the classical framework described above: We take the posterior distribution of ***δ*** to be approximately normally distributed with mean equal to the classical estimate and covariance equal to *s* times the corresponding classical covariance matrix of the estimated effects, where we use the prior means (instead of the point estimates) of the unknown variance components in this classical analysis. The reason for choosing this proxy distribution for ***δ*** is that we know the classical and Bayesian inferences for the basic parameters will in general be in reasonable agreement, but also that standard classical analyses, by ignoring the uncertainty in the unknown variance components, will provide artificially small standard errors. See the examples of Jackson et al. [7] for demonstrations of this. By using the prior means of the unknown variance components in the classical estimation that underlies the motivation for the proxy distribution for ***δ***, we continue to assume that there is relatively little information about the unknown variance components in the sample. In large samples, where there is more information about these parameters, we might therefore prefer to use point estimates of $\tau ^{2}_{\beta }$ and $\tau ^{2}_{\omega }$ in the classical estimation that underlies the proxy distribution for ***δ***.

Finally, we require *s*≥1 to ensure that the proxy distribution has heavier tails than the posterior distribution, and to overcome the tendency of the standard classical analysis to understate the uncertainty in the basic parameters compared to the Bayesian analysis. We follow Turner et al. [19] and use *s*=4 for this purpose, which is a suitably large value that compensates for artificially small classical standard errors that are only half their Bayesian counterparts (posterior standard deviations). To summarise, (6) is an intuitively appealing proxy distribution that can be expected to have heavy tails but otherwise resemble the posterior distribution quite well.

As stated above, the simulated samples from the proxy distribution are weighted by the ratio of the densities of the true distribution to the proxy distribution, evaluated at the simulated values. Here the true distribution is the correct posterior distribution implied by the Bayesian analysis and the proxy is given in (6). The weights are therefore given by

(7) $$  w^{*}_{i} = \frac{f_{{MVN}} \left(\mathbf{Y}, \mathbf{X} \boldsymbol{\delta}^{*}_{i}, \mathbf{Y}_{{var}} \right)}{f_{{MVN}} \left(\boldsymbol{\delta}^{*}_{i}, \: \widetilde{\boldsymbol{\delta}}, \: s \, \boldsymbol{\widetilde{\delta}}_{{var}}\right)},  $$

where *f*_*MVN*_(·,***μ***,***Σ***) is the probability density function of the multivariate normal distribution with mean ***μ*** and covariance matrix ***Σ*** and $ \mathbf {Y}_{{var}} = \tau ^{2*}_{\beta } \mathbf {M}_{1} + \tau ^{2*}_{\omega } \mathbf {M}_{2} + \mathbf {S}.$ The weights given in (7) are a direct extension of the importance sampling weights for conventional meta-analysis given by Turner et al. [19]. Briefly, the constant of proportionality in the Bayesian analysis and the constant due to the uniform priors for the basic parameters are common to all weights and so are ignored in (7); strictly speaking, our formula for weights describes relative weights, but relative weighting is all that is required. The fact that the constant of proportionality is common to all weights, and so need not be evaluated, is the reason why importance sampling is useful in Bayesian analysis. The lognormal distributions are common to the numerator and denominator of (7) and so cancel: they appear in the numerator because the posterior distribution is proportional to the prior, and they appear in the denominator because they are used in the proxy distribution (6). Hence, after cancellation, all that remains in the numerators of the weights is the likelihood evaluated at the simulated values, which is $f_{{MVN}} \left (\mathbf {Y}, \mathbf {X} \boldsymbol {\delta }^{*}_{i}, \mathbf {Y}_{{var}}\right)$, and all that remains in the denominators is the density of the proxy distribution of ***δ***^∗^ evaluated at the simulated values, which is $f_{{MVN}} \left (\boldsymbol {\delta }^{*}_{i}, \: \widetilde {\boldsymbol {\delta }}, \: s \, \boldsymbol {\widetilde {\delta }}_{{var}}\right)$. In principle, any heavy tailed proxy distribution could be used instead of (6) but we see that the proxy distribution is both intuitively appealing and results in a very simple formula for the weights. Since the weights depend on the simulated values from the proxy distribution, they are random and so we use an asterisk in their notation.

Weighted samples from the proxy distribution can then be used to obtain any numerical summary of the posterior distribution, including parameter estimates, moments, and quantiles. For example, the posterior mean of the between-study heterogeneity variance $\tau ^{2}_{\beta }$ may be estimated from the simulated values of $\tau ^{2*}_{\beta i}$ as

$$\widehat{\tau}^{2}_{\beta} = \frac{\sum\limits_{i=1}^{n} \tau^{2*}_{\beta i} w^{*}_{i}}{\sum\limits_{i=1}^{n} w^{*}_{i}}, $$

where *n* is the number of simulated values from the proxy distribution. Monte Carlo errors of the estimated posterior means, which we will present as estimates from the Bayesian analyses, can be obtained in the way proposed by Turner et al. [19] in their supplementary materials. The full importance sampling code, and the data for both examples, is also provided in the Additional file 4. We also provide OpenBUGS code for both examples in the Additional file 5, so that the results obtained using importance sampling and MCMC can be compared. This code is taken from Jackson et al. [8] with trivial changes to the prior specification. Since these methods implement the same Bayesian analysis, they should be, to within Monte Carlo error, in numerical agreement. This is the case for both examples and together the two approaches to Bayesian computation provide a convenient way to confirm the accuracy of the numerical algorithms.

### Likelihood-based methods

The Bayesian methods described above require the specification of prior distributions for all parameters. In particular, they require priors for the unknown variance components. A classical analysis makes fewer assumptions because it does not require the specification of prior distributions. As explained above, the standard method for making inferences about the average treatment effects in the classical framework initially estimates the unknown variance components and then treats them as known. Analysis is then straightforward. However, the only classical method that has previously been proposed for estimating the unknown variance components in the model given by (2) is based on the method of moments [7]. Although this method is semi-parametric (in the sense of making no distributional assumptions as part of the estimation procedure) and computationally undemanding, it is not based on sufficient statistics and so is not fully efficient.

Likelihood-based methods are based on sufficient statistics and are fully efficient in large samples. In the Additional file 5, we provide R code for use with the *metafor* package [23], that makes use of the *rma.mv* command to fit the model using likelihood-based methods. Briefly, model (2) implies a (log) likelihood function that depends on the parameters in ***δ*** and the two variance components $\tau ^{2}_{\beta }$ and $\tau ^{2}_{\omega }$. However, for given values of the variance components, the maximum likelihood estimate (MLE) of ***δ*** is given by (3), and therefore model fitting reduces to an optimization problem that involves maximizing the (log) likelihood over the two parameters $\tau ^{2}_{\beta }$ and $\tau ^{2}_{\omega }$. This can be achieved using standard numerical procedures, such as quasi-Newton or Nelder-Mead type algorithms [24]. Once the MLEs of the variance components are found (and hence the MLE of ***δ***), the asymptotic sampling variance of $\boldsymbol {\hat {\delta }}$ is given by (4) and inference about ***δ*** can proceed as described earlier.

However, we follow the defaults of *rma.mv* and use restricted maximum likelihood (REML) estimation instead, although this can be changed by the analyst if desired. In essence, REML uses a modification of the usual likelihood in order to compensate for the fact that maximum likelihood estimates of variance components tend to be biased downward [18]. Therefore, in the examples below, we use the *rma.mv* command to obtain the REML estimates of $\vspace *{-.5pt}\tau ^{2}_{\beta }$ and $\tau ^{2}_{\omega }$, which are then used as described above to make inferences about the average treatment effects. In the Additional file 5, we also show how the *rma.mv* command can be used to fit a consistency model, where we assume that $\tau ^{2}_{\omega }=0$, and compare the models with and without the consistency assumption, using the likelihood ratio test.

There are further advantages to using a likelihood-based approach for estimating the unknown variance components. First, the asymptotic theory of maximum likelihood provides a straightforward way to obtain approximate confidence intervals and hypothesis tests for all parameters, including the unknown variance components. Below, we obtain confidence intervals for the variance parameters using the profile likelihood method, extending previous applications thereof in the context of the standard random-effects model [25]. Obtaining these types of inferences for the unknown variance components when estimating them using the method of moments is more difficult, and at present some form of bootstrapping is most likely required for this purpose [7].

Second, by using likelihood-based methods, the estimation procedure for alternative models (that could, for example, involve more complicated assumptions for the between-study variance or inconsistency variance components) is immediate, although a challenge is still the implementation and fine-tuning of the increasingly complex numerical procedures required for obtaining the ML/REML estimates. Conversely, when using the method of moments, alternative estimation procedures are needed for different types of model. Hence, the way to develop estimation procedures that use the method of moments for more complicated models involving additional variance components is not so obvious.

The likelihood-based results we obtain below for both examples that follow are in good agreement with other methods. The practical implementation of likelihood-based estimation methods for models for network meta-analysis, and the accompanying code for this purpose, is one of the main contributions of this paper.

### Other software

We implement our methods in R, and compare them to results obtained using MCMC in BUGS software. However, our methods are not restricted to this software. In principle, these methods could be implemented in SAS, using PROC GLIMMIX, or in Stata. Existing Stata packages for undertaking network meta-analysis using existing methods include *network* [26] and *network graphs* [27]. Further, MCMC methods may be undertaken in SAS using PROC MCMC.

## Results and discussion

In this section we apply our two new methods (Bayesian estimation using importance sampling and informative priors, and REML estimation) to two contrasting examples and discuss the results obtained. For the importance sampling method, the analysis was undertaken using three different prior distributions for inconsistency: using a lognormal prior that is identical to the prior for the between-study heterogeneity variance; using a lognormal prior distribution with an expected value of half the prior mean for heterogeneity, and using a lognormal prior with an expected value of one tenth the prior mean for heterogeneity. We present the results for the second of these possibilities for the prior distribution of $\tau ^{2}_{\omega }$ in the main paper, while the results for the other prior distributions for $\tau ^{2}_{\omega }$ can be found in the Additional file 5. When using MCMC, we used a single chain with a burn in of 100,000, and 1,000,000 iterations to make inferences. We appreciate that multiple chains may be run as a check of convergence; however, here, a comparison with the results obtained using importance sampling provided satisfactory evidence of convergence. When using importance sampling, no burn in is required and we used 1,000,000 iterations to make inferences. This number of iterations was chosen to minimise MC error to compare the methods.

The Bayesian analyses using MCMC were implemented in OpenBUGS, an open source version of WinBUGS [9]. All other computations were performed using R [28]. Bayesian analyses using importance sampling and MCMC were implemented using the R and OpenBUGS code provided in the Additional file 3. All importance sampling and MCMC inferential output for the examples are, to within Monte Carlo error, in numerical agreement with each other. Since both the MCMC and importance sampling methods fit the same Bayesian model, checking this agreement is in essence a check of the convergence of the two methods. For all Bayesian analyses, we present posterior means as estimates, but also include quantiles as the posterior distribution of variance components is generally skew. The results for the likelihood-based estimation method (REML) were obtained in R using the package *metafor* [23], based on the code provided in the Additional file 5.

### Example one: prostate cancer

The first example dataset was collected and previously analysed by Xiong et al. [29], who evaluate the comparative efficacy and safety of a number of treatments for prostate cancer. The dataset comprises seventeen two-arm studies that compare eight categories of treatments: A – Observational management (reference treatment); B – prostatectomy; C – conventional radiotherapy (CR); D – conventional radiotherapy hypofractionated (CR-HF); E – 3D conformal low dose radiotherapy (CF-LD); F – 3D conformal high dose radiotherapy (CF-HD); G – 3D conformal low dose hypofractionated (CF-LD-HF); and H – cryotherapy. The authors examined two measures of efficacy, all-cause mortality and prostate cancer-caused mortality; we have examined all-cause mortality only, using the log odds ratio as the measure of treatment effect. As all-cause mortality is an adverse outcome and we use the log odds ratio, a negative treatment effect indicates that the active treatment performs better than the reference treatment.

The priors we used in the Bayesian analysis presented in the main body of this paper are$\tau ^{2}_{\beta } \sim LN(-3.50, 1.26^{2})$ and $\tau ^{2}_{\omega } \sim LN(-4.80, 1.68^{2})$, so that the lognormal prior distribution for the inconsistency variance has an expected value of half of that of the prior distribution for the between-study heterogeneity variance. This represents the position that inconsistency is to be anticipated in a network meta-analysis but it would not usually be expected to be too severe. The estimates obtained using a Bayesian analysis with these informative prior distributions, implemented using both importance sampling and OpenBUGS, are shown in Table 1, and are in approximate agreement with those from Xiong et al. [29] (see their Table 1, row 1), including the inferences concerning the magnitude of the between-study heterogeneity.

**Table 1:** Results for the prostate cancer data using importance sampling, MCMC in OpenBUGS and REML estimation. Estimates are log odds ratios

		Parameter	Estimate	SD	MCSE	2.5 %	50 %	97.5 %
Importance sampling	B: Prostatectomy	*δ* ^*AB*^	-0.25	0.19	0.0015	-0.63	-0.25	0.11
	C: CR	*δ* ^*AC*^	-0.16	0.34	0.0033	-0.82	-0.15	0.50
	D: CR-HF	*δ* ^*AD*^	-0.32	0.41	0.0038	-1.13	-0.32	0.49
	E: CF-LD	*δ* ^*AE*^	-0.31	0.30	0.0030	-0.90	-0.31	0.27
	F: CF-HD	*δ* ^*AF*^	-0.35	0.35	0.0033	-1.02	-0.35	0.33
	G: CF-HF-LD	*δ* ^*AG*^	-0.66	0.87	0.0085	-2.34	-0.67	1.07
	H: Cryotherapy	*δ* ^*AH*^	-0.27	0.53	0.0051	-1.32	-0.29	0.80
	Heterogeneity	$\tau ^{2}_{\beta }$	0.02	0.02	0.0002	0.00	0.01	0.08
	Inconsistency	$\tau ^{2}_{\omega }$	0.02	0.04	0.0002	0.00	0.01	0.11
MCMC	B: Prostatectomy	*δ* ^*AB*^	-0.25	0.19	0.0011	-0.63	-0.24	0.12
	C: CR	*δ* ^*AC*^	-0.16	0.34	0.0036	-0.83	-0.16	0.51
	D: CR-HF	*δ* ^*AD*^	-0.32	0.42	0.0039	-1.14	-0.32	0.50
	E: CF-LD	*δ* ^*AE*^	-0.31	0.30	0.0030	-0.90	-0.31	0.28
	F: CF-HD	*δ* ^*AF*^	-0.35	0.35	0.0036	-1.03	-0.35	0.34
	G: CF-HF-LD	*δ* ^*AG*^	-0.63	0.86	0.0100	-2.33	-0.64	1.06
	H: Cryotherapy	*δ* ^*AH*^	-0.27	0.54	0.0048	-1.32	-0.27	0.78
	Heterogeneity	$\tau ^{2}_{\beta }$	0.02	0.02	0.0001	0.00	0.01	0.08
	Inconsistency	$\tau ^{2}_{\omega }$	0.02	0.04	0.0003	0.00	0.01	0.11
		Parameter	Estimate	SE	CI lower	CI upper	*p*-value	
REML estimation	B: Prostatectomy	*δ* ^*AB*^	-0.22	0.11	-0.43	-0.01	0.04	
	C: CR-CF	*δ* ^*AC*^	-0.17	0.28	-0.72	0.39	0.56	
	D: CR-HF	*δ* ^*AD*^	-0.33	0.32	-0.96	0.31	0.31	
	E: CF-LD	*δ* ^*AE*^	-0.32	0.25	-0.80	0.17	0.21	
	F: CF-HD	*δ* ^*AF*^	-0.35	0.28	-0.90	0.19	0.20	
	G: CF-HF-LD	*δ* ^*AG*^	-0.65	0.83	-2.27	0.97	0.43	
	H: Cryotherapy	*δ* ^*AH*^	-0.28	0.47	-1.20	0.65	0.56	
	Heterogeneity	$\tau ^{2}_{\beta }$	0.00	–	0.00	0.07	–	
	Inconsistency	$\tau ^{2}_{\omega }$	0.00	–	0.00	0.62	–	

*CR*=conventional radiotherapy; *HF*=hypofractionated; *CF*=3D conformal; *LD*=low dose; *HD*=high dose

**Table 2:** Results for the topical antibiotics data using importance sampling, MCMC in OpenBUGS and REML estimation. Estimates are log odds ratios

		Parameter	Estimate	SD	MCSE	2.5 %	50 %	97.5 %
Importance	B: Quinolone	*δ* ^*AB*^	-1.85	0.62	0.0012	-3.18	-1.82	-0.69
sampling	C: Non-quinolone	*δ* ^*AC*^	-1.31	0.71	0.0014	-2.78	-1.29	0.03
	D: Antiseptic	*δ* ^*AD*^	-0.64	0.65	0.0013	-2.01	-0.62	0.60
	Heterogeneity	$\tau ^{2}_{\beta }$	0.28	0.31	0.0006	0.01	0.18	1.09
	Inconsistency	$\tau ^{2}_{\omega }$	0.22	0.38	0.0007	0.00	0.08	1.20
MCMC	B: Quinolone	*δ* ^*AB*^	-1.85	0.62	0.0052	-3.15	-1.82	-0.68
	C: Non-quinolone	*δ* ^*AC*^	-1.30	0.71	0.0061	-2.77	-1.28	0.04
	D: Antiseptic	*δ* ^*AD*^	-0.64	0.65	0.0053	-1.99	-0.62	0.61
	Heterogeneity	$\tau ^{2}_{\beta }$	0.28	0.31	0.0017	0.01	0.18	1.09
	Inconsistency	$\tau ^{2}_{\omega }$	0.22	0.38	0.0030	0.00	0.08	1.19
		Parameter	Estimate	SE	CI lower	CI upper	*p*-value	
REML	B: Quinolone	*δ* ^*AB*^	-1.97	0.68	-3.30	-0.65	<0.01	
estimation	C: Non-quinolone	*δ* ^*AC*^	-1.40	0.81	-2.98	0.18	0.08	
	D: Antiseptic	*δ* ^*AD*^	-0.66	0.72	-2.07	0.75	0.36	
	Heterogeneity	$\tau ^{2}_{\beta }$	0.10	–	0.00	1.67	–	
	Inconsistency	$\tau ^{2}_{\omega }$	0.54	–	0.00	3.96	–	

The estimates found using the likelihood-based method are also shown in Table 1. The results are the same as those from the consistency model (where $\tau ^{2}_{\omega }=0$) as the inconsistency variance estimate is zero. Furthermore, the REML results agree exactly with the results obtained using the method of moments [7] because both the REML and the moments-based estimates of both variance components are zero. Hence for this example, the classical analyses collapse to a common-effects analysis, where $\hat {\tau }^{2}_{\beta }=\hat {\tau }^{2}_{\omega }=0$. In the Additional file 3, the likelihood ratio test shows no difference between the models because $\hat {\tau }^{2}=0$. The Bayesian analysis also reflects the absence of between-study variation and inconsistency, because the Bayesian estimates of these parameters are both close to zero. However, the prior distributions for these parameters pull the posterior means slightly away from zero.

Interestingly, the 95 % credibility interval for $\tau ^{2}_{\beta }$ based on the Bayesian analyses (i.e., 0.00 to 0.08) is in close agreement with the 95 % profile likelihood confidence interval based on the likelihood-based analysis (i.e., 0.00 to 0.07). Since the latter does not incorporate any prior information into the analysis, this suggests that inferences about the amount of heterogeneity are mostly driven by the information contained in the data at hand. On the other hand, the credibility interval for $\tau ^{2}_{\omega }$ (i.e., 0.00 to 0.11) is substantially narrower than the confidence interval (i.e., 0.00 to 0.62). Therefore, relative to the amount of information contained in the data about the degree of inconsistency in the network, the prior for $\tau ^{2}_{\omega }$ is exerting a noticeable influence on the results of this analysis.

Also, while the Bayesian and classical inferences for the basic parameters are in good agreement, the Bayesian credible intervals are a little wider than the classical confidence intervals. This is often the case in practice, because the Bayesian analyses allow for the uncertainty in the unknown variance components [7].

When the prior distribution for the inconsistency is varied, the heterogeneity estimate remains similar ($\hat {\tau }^{2}_{\beta } = 0.02$), while the inconsistency estimate decreases as the expected mean of the prior is decreased (mean equal to heterogeneity prior mean: $\hat {\tau }^{2}_{\omega } = 0.04$; one half: $\hat {\tau }^{2}_{\omega } = 0.02$; one tenth: $\hat {\tau }^{2}_{\omega } = 0.00$, all in Additional files 1 and 4). The choice of inconsistency prior is influential, which is to be expected as we are estimating nine parameters (the seven basic parameters and two variance components) using seventeen comparisons.

To summarise, our analyses are in reasonable agreement and suggest that there is little between-study heterogeneity or inconsistency in the study results. This in turn suggests that the true underlying treatment effects are very similar in all studies in the population. The analyses also suggest that all eight treatments have a similar average effect. Hence the overall picture from our analysis is that, in terms of treatment efficacy for the outcome of all-cause mortality, there is little to choose between the treatments in any study.

### Example two: topical antibiotics

The second set of example data is from a Cochrane Systematic Review entitled “Topical antibiotics without steroids for chronically discharging ears with underlying eardrum perforations” [30]. The dataset comprises thirteen studies (three three-arm studies, ten two-arm studies) and compares three pharmacological treatments (B – topical quinolone antibiotic; C – topical non-quinolone antibiotic; and D – topical antiseptic) to no treatment (A). This dataset was analysed by Jackson et al. [7] using the same model as used in this paper, but using the method of moments. As the treatment effects are log odds ratios comparing the other treatments to treatment *A*, and the outcome is harmful (treatment failure due to persistent discharge), negative basic parameters indicate that treatments B, C and D are more beneficial than treatment A (no treatment).

The priors we used in the Bayesian analysis presented in the main paper are $\tau ^{2}_{\beta } \sim LN(-2.29, 1.58^{2})$ and $\tau ^{2}_{\omega } \sim LN(-3.64, 1.95^{2})$. Although one of the treatments, treatment A (“no treatment") is not pharmacological, we make the pragmatic decision to use a prior distribution for heterogeneity for pharmacological vs. pharmacological intervention comparisons. The Bayesian results are shown in Table 2 and and are set out in a similar way as the table for the first example. The estimates of the treatment effects are similar to those shown in Jackson et al. [7] (see their Table 4). The estimates of the between-study and inconsistency variances in Table 2 are smaller than the corresponding Bayesian estimates in Jackson et al. ($\hat {\tau }^{2}_{\beta } = 0.56$, $\hat {\tau }^{2}_{\omega } = 0.96$), where vague uniform priors from 0 to 5 were placed on *τ*_*β*_ and *τ*_*ω*_, but are still large enough to be described as high. This suggests that there is considerable between-study variation and also inconsistency in treatment effect estimates across designs. Furthermore, the posterior standard deviations of the basic parameters in Table 2 are also smaller than those of the previous Bayesian analyses [7]. Bayesian credible intervals are often wider than the corresponding classical confidence intervals, and the smaller posterior standard deviations (and resulting narrower credible intervals) in comparison to the classical results in this example can be explained by the use of informative priors. The informative priors therefore have considerable impact in this example. Full results from the analysis using uniform priors are available in Jackson et al., [7] (their Table 4).

The estimates found using the likelihood-based method are also shown in Table 2. Again we infer that considerable inconsistency is present ($\hat {\tau }^{2}_{\omega } = 0.54$), so this time the results differ from those that are obtained using the corresponding consistency model. Comparing the model fits using the likelihood ratio test shows no statistically significant evidence of an improvement in model fit. The estimated between-study heterogeneity variance is also larger than zero ($\hat {\tau }^{2}_{\beta } = 0.10$), but not as large as the corresponding estimate using the method of moments [7]. However, the sums of the REML- and moment-based point estimates of the variance components (i.e., $\hat {\tau }^{2}_{\beta } + \hat {\tau }^{2}_{\omega }$) are in better agreement. Since the marginal variance of any entry of **Y** is equal to $\tau ^{2}_{\beta }+\tau ^{2}_{\omega }$ plus the corresponding within-study variance, we would anticipate this type of agreement. However, the REML analysis attributes more of the total variation in the data to inconsistency than the previous moment-based analysis. The new results obtained here, when compared to those reported previously, nicely illustrate the difficulty in separating the two unknown variance components in small samples. If desired, a user could additionally perform interval estimation for the sum of the variance components, with the intuition that this sum will be better identified than the individual variance components.

The relevance of the informative priors for $\tau ^{2}_{\beta }$ and $\tau ^{2}_{\omega }$ also becomes quite apparent when we compare again the 95 % credibility and confidence intervals for these parameters. Specifically, the profile likelihood confidence interval for $\tau ^{2}_{\omega }$ is very wide (i.e., 0.00 to 3.96), while the credibility interval is much narrower (0.00 to 1.20 or 1.19, for the importance sampling and MCMC, respectively). The latter is based not only on the information contained in the data about the degree of inconsistency in the network, but also the information contained in the prior distribution. In fact, the 2.5th and 97.5th percentiles of the lognormal prior distribution for $\tau ^{2}_{\omega }$ are 0.03 and 1.20, respectively, so the inferences about this parameter in the Bayesian analyses appear to be driven to a large extent by what was specified via the prior. This is further shown by noting the estimates of the variance components when using different informative priors: When the expected mean of the inconsistency prior is equal to that of the heterogeneity prior, the estimates obtained using importance sampling (and MCMC) are $\hat {\tau }^{2}_{\beta } = 0.22, \hat {\tau }^{2}_{\omega } = 0.35$. When the expected mean is one tenth, the estimates are $\hat {\tau }^{2}_{\beta } = 0.37, \hat {\tau }^{2}_{\omega } = 0.06$ (see Additional files 1 and 4). As with the first example, the choice of inconsistency prior is influential; in this instance, we are estimating six parameters using sixteen comparisons.

With respect to the basic parameters, inferences based on the Bayesian analyses, the likelihood-based analysis, and the method of moments are all in relatively good agreement, with quinolone yielding the largest point estimate of treatment effect, which we also infer to be more effective than no treatment.

## Conclusions

We have presented two new methods for fitting models for network meta-analyses that include random inconsistency effects. Both methods are described in order to fit an existing model for network meta-analysis. The first new method implements a Bayesian analysis using importance sampling and informative lognormal prior distributions for the unknown variance components. The second new method uses likelihood-based methods, and in particular REML estimation, to fit the model. Although importance sampling and REML estimation are not methodologically new, to our knowledge this is the first time these methods have been applied in this particular setting.

Although we present our methods in terms of the model proposed by Jackson and colleagues, we can modify the model to allow different forms of inconsistency variance components by using a different matrix **M**_2_. For example, by modifying this matrix we can describe loop inconsistency models [7] and also other types of models that use random-inconsistency effects. Hence our methods are very easily modified to fit alternative models: one simply replaces **M**_2_ with a different matrix and proceeds as described above. We assume random inconsistency effects so that the basic parameters can be interpreted as average treatment effects across the network, so that ‘the end justifies the means’. However, the case for using fixed inconsistency effects instead can also be made, mainly on the grounds that the distributional assumptions made when using random inconsistency effects are strong and may be unrealistic [12].

The model could also be extended with further variance parameters, by introducing more products of **M** matrices with corresponding variance components. For example, Gumedze and Jackson [31] describe a random effects variance shift model for detecting and accommodating outliers in conventional univariate meta-analyses. The same idea could be applied in the present context, by adding random shift effects for particular contrasts, studies, and/or designs.

We have focused on estimation in this paper. However, alternative forms of inference, such as treatment ranking, *I*^2^ statistics, prediction intervals, model diagnostics, and so on are also very important and are likely to form the subject of future work. The focus of our attention on the two new estimation methods developed here should not be taken to mean that other inferential aspects should be ignored. Rather we emphasise estimation in this paper because this is the aspect of the methodology for network meta-analysis that this work is intended to extend.

The model provides a way of estimating inconsistency in a network meta-analysis. When it is clear that inconsistency is considerable, we would strongly discourage proceeding with a network meta-analysis. One might choose instead to re-evaluate the criteria for study inclusion, or examine where the inconsistency occurs [10]. When there is strong evidence that inconsistency is low, one might consider making the consistency assumption. However, we would still recommend using a random-effects model. When information regarding inconsistency is low and its estimate may be dominated by its prior, a sensitivity analysis may be undertaken: a range of priors may be used, or the results may be compared to a variety of frequentist analyses.

This work extends the methodology of using informative priors for the between-study heterogeneity variance to also using informative priors for the inconsistency variance. To our knowledge, informative priors have not previously been used to inform the estimation of inconsistency parameters in network meta-analysis. As possible future work, data-based predictive distributions for the inconsistency variance in network meta-analysis in various settings could be produced in an analogous manner as that undertaken by Turner et al. [19] for the between-study heterogeneity. We have chosen informative priors for inconsistency that have an expected mean of one half that of the between-study heterogeneity, and used other ratios as a form of sensitivity analysis. However, other approaches could be considered. For example, further empirical work could investigate the relationship between between-study heterogeneity and inconsistency, possibly by placing vague priors on both and examining the ratio of estimates in various settings.

The use of Bayesian methods facilitates making statements about the uncertainty in the estimated between-study heterogeneity variance and the inconsistency variance, which is preferable to reporting point estimates only [32]. Likelihood-based methods can also be used to obtain corresponding confidence intervals, but Bayesian methods also automatically take the uncertainty in these variance components into account when making inferences about the average treatment effects. However, this benefit of the Bayesian approach comes at the cost of making more assumptions via the prior distributions. For example, we make repeated use of the prior knowledge afforded by Turner et al. [19] (because we use it to form two prior distributions) without recognising that fact in the analysis. Another criticism is that, since inconsistency in network meta-analysis is less well understood than between-study variation, it is quite reasonable to assert that we have less prior knowledge about $\tau ^{2}_{\omega }$ than $\tau ^{2}_{\beta }$, so that the prior distribution of the former unknown variance should be more diffuse than the prior distribution of the latter one. This could be taken into account by scaling *V* by a factor of greater than one before using (5) to derive the prior for $\tau ^{2}_{\omega }$. For now, we are content to use our suggested procedure as a pragmatic way to derive informative prior distributions for the inconsistency variance. We would be pleased to replace this procedure for obtaining informative priors for $\tau ^{2}_{\omega }$ with priors derived using a modelling approach like that of Turner et al. [19], if and when they become available.

An advantage of a Bayesian approach is flexibility: For example, they allow us to take exact likelihood approaches and specify non-normal random-effects distributions if desired. Further, future work could extend the presented methods to allow the specification of the binomial within-study likelihood, which is preferable for binary data. Both Bayesian and classical methods have their advantages and disadvantages, and so we present estimation methods in both of these conceptual frameworks.

The full computing code for both new methods is included in the Additional file 2, accompanied by the two working examples described above. In the case that a likelihood-based approach is preferred, models can be fit within an existing package. This feature of the *metafor* package has never been presented before. Both new estimation methods have been found to give results comparable to those obtained using existing methods in our two contrasting examples.

Implementing Bayesian analyses using both MCMC and importance sampling, as we have done, is a practical way to verify Bayesian analyses that use MCMC methods. We recommend that authors consider this in application.

In summary, we have developed two new estimation methods for models for network meta-analysis with random inconsistency effects. Our new Bayesian methodology can be undertaken without recourse to MCMC or the Win/OpenBUGS software and our new classical methodology means that the *metafor* package can now be used to fit models of this type. We hope that this paper and the accompanying computing code will help to make network meta-analysis more accessible to applied researchers.

## Additional files

Additional file 1 R code. (DOCX 11 kb)

Additional file 2 Matrices M _***1***_ and M _***2***_ from example 2: Topical antibiotics. (PDF 98 kb)

Additional file 3 Difference between R and BUGS results. (DOCX 5 kb)

Additional file 4 OpenBUGS code. (DOCX 41 kb)

Additional file 5 Fitting Jackson’s model using metafor. (DOCX 31 kb)

## References

[1] DerSimonian R, Laird N. Meta-analysis in clinical trials. Control Clin Trials. 1986; 7:177–88. 10.1016/0197-2456(86)90046-2 3802833

[2] Salanti G. Indirect and mixed-treatment comparison, network, or multiple-treatments meta-analysis: many names, many benefits, many concerns for the next generation evidence synthesis tool. Res Synth Methods. 2012; 3:80–97. 10.1002/jrsm.1037 26062083

[3] Salanti G, Higgins JPT, Ades AE, Ioannidis JPA. Evaluation of networks of randomized trials. Stat Methods Med Res. 2008; 17:279–301. 10.1177/0962280207080643 17925316

[4] Dias S, Ades AE (2016). Absolute or relative effects? Arm-based synthesis of trial data. Res Synth Methods.

[5] Hong H, Chu H, Zhang J, Carlin BP (2016). Rejoinder to the discussion of “a Bayesian missing data framework for generalized multiple outcome mixed treatment comparisons,” by S. Dias and A.E. Ades. Res Synth Methods.

[6] Piepho HP, Williams ER, Madden LV. The use of two-way linear mixed models in multitreatment meta-analysis. Biometrics. 2012; 68:1269–77. 10.1111/j.1541-0420.2012.01786.x 22845838

[7] Jackson D, Law M, Barrett JK, Turner R, Higgins JPT, Salanti G, White IR (2016). Extending DerSimonian and Laird’s methodology to perform network meta-analysis with random inconsistency effects. Stat Med.

[8] Jackson D, Barrett JK, Rice S, White IR, Higgins JPT. A design-by-treatment interaction model for network meta-analysis with random inconsistency effects. Stat Med. 2014; 33:3639–54. 10.1002/sim.6188 PMC4285290 24777711

[9] Lunn DJ, Thomas A, Best N, Spiegelhalter D. WinBUGS– a Bayesian modelling framework: concepts, structure, and extensibility. Stat Comput. 2000; 10:325–37.

[10] Krahn U, Binder H, König J. A graphical tool for locating inconsistency in network meta-analyses. BMC Med Res Methodol. 2013; 13:35. 10.1186/1471-2288-13-35 PMC3644268 23496991

[11] Lambert PC, Sutton AJ, Burton PR, Abrams KR, Jones DR. How vague is vague? A simulation study of the impact of the use of vague prior distributions in MCMC using WinBUGS. Stat Med. 2005; 24:2401–28. 10.1002/sim.2112 16015676

[12] White IR, Barrett JK, Jackson D, Higgins JPT. Consistency and inconsistency in network meta-analysis: model estimation using multivariate meta-regression. Res Synth Methods. 2012; 3:111–25. 10.1002/jrsm.1045 PMC4433771 26062085

[13] Higgins JPT, Jackson D, Barrett JK, Lu G, Ades AE, White IR. Consistency and inconsistency in network meta-analysis: concepts and models for multi-arm studies. Res Synth Methods. 2012; 3:98–110. 10.1002/jrsm.1044 PMC4433772 26062084

[14] Lu G, Ades AE. Assessing evidence inconsistency in mixed treatment comparisons. J Am Stat Assoc. 2006; 101:447–59.

[15] Jackson D, Boddington P, White IR. The design-by-treatment interaction model: a unifying framework for modelling loop inconsistency in network meta-analysis. Res Synth Methods. 2015. doi:http://dx.doi.org/10.10021jrsm1188. 10.1002/jrsm.1188 PMC4946625 26588593

[16] Piepho HP. Network meta-analysis made easy: detection of inconsistency using factorial analysis-of-variance models. BMC Med Res Methodol. 2014; 14:61. 10.1186/1471-2288-14-61 PMC4049370 24885590

[17] Lumley T. Network meta-analysis for indirect treatment comparisons. Stat Med. 2002; 21:2313–24. 10.1002/sim.1201 12210616

[18] Jackson D, Riley R, White IR. Multivariate meta-analysis: Potential and promise. Stat Med. 2011; 30:2481–98. 10.1002/sim.4172 PMC3470931 21268052

[19] Turner RM, Jackson D, Wei Y, Thompson SG, Higgins JPT. Predictive distributions for between-study heterogeneity and methods for their application in Bayesian meta-analysis. Stat Med. 2014; 34:984–98. 10.1002/sim.6381 PMC4383649 25475839

[20] Whitehead A (2002). Meta-analysis of Controlled Clinical Trials.

[21] Kendall FJ, O’Hagan (2004). Advanced Theory of Statistics, vol 2B.

[22] Baker R, Jackson D. A new approach to outliers in meta-analysis. Health Care Manag Sci. 2008; 11:121–31. 10.1007/s10729-007-9041-8 18581818

[23] Viechtbauer W. Conducting meta-analyses in R with the metafor package. J Stat Softw. 2010; 36:1–48. http://www.jstatsoft.org/v36/i03/. Accessed 19 July 2016.

[24] Nocedal J, Wright SJ (1999). Numerical Optimization.

[25] Hardy RJ, Thompson SG. A likelihood approach to meta-analysis with random effects. Stat Med. 1996; 15:619–29. 10.1002/(SICI)1097-0258(19960330)15:6<619::AID-SIM188>3.0.CO;2-A 8731004

[26] White IR (2015). Network meta-analysis. Stata J.

[27] Chaimani A, Salanti G (2015). Visualizing assumptions and results in network meta-analysis: The network graphs package. Stata J.

[28] R Core Team. R: A language and environment for statistical computing. Vienna, Austria: R Foundation for Statistical Computing. http://www.R-project.org/. Accessed 19 July 2016.

[29] Xiong T, Turner R, Wei Y, Neal DE, Lyratzopoulos G, Higgins JPT. Comparative efficacy and safety of treatments for localized prostate cancer: an application of network meta-analysis. BMJ Open. 2014; 4:e004285. 10.1136/bmjopen-2013-004285 PMC4024605 24833678

[30] Macfadyen CA, Acuin JM, Gamble C. Topical antibiotics without steroids for chronically discharging ears with underlying eardrum perforations. Cochrane Database Syst Rev. 2005; 4:CD004618. 10.1002/14651858.CD004618.pub2 PMC6669264 16235370

[31] Gumedze FN, Jackson D. A random effects variance shift model for detecting and accommodating outliers in meta-analysis. BMC Med Res Methodol. 2011; 11:19. 10.1186/1471-2288-11-19 PMC3050872 21324180

[32] Jackson D, Turner R, Rhodes K, Viechtbauer W. Methods for calculating confidence and credible intervals for the residual between-study variance in random effects meta-regression models. BMC Med Res Methodol. 2014; 14:103. 10.1186/1471-2288-14-103 PMC4160560 25196829

