# Supplementary material for: Two new methods to fit models for network meta-analysis with random inconsistency effects
Source: BMC Med Res Methodol. 2016 Jul 28;16:87. doi: 10.1186/s12874-016-0184-5 (PMC4964019; doi:10.1186/s12874-016-0184-5)
Supplement: Additional file 1 — R code. (DOCX 11 kb) [file 12874_2016_184_MOESM1_ESM.docx]

**Supplementary material: Estimating inconsistency in network meta-analysis using importance sampling**

**R code**

The R function ismeta estimates treatment effects in our network meta-analysis model using importance sampling. The code notation closely follows that of the main paper. The function takes the following arguments:

sims: the number of iterations to run.

all_data: a list containing the data. The list must be comprised of the following: Two further lists, y and s, and the vector d, all of length equal to the total number of studies. The list y contains the estimated treatment effects *y_d_*_i_, which are scalars if there is only one effect (e.g. design *BC*), vectors otherwise. The list s contains the within-study covariance matrices *s_di_*, which again may be scalars if there is only one effect. The vector d contains the design of each study, (e.g. *AB*, *AC*, *BDE*). Each treatment should be allocated a letter, with *A* being the reference treatment.

mu.b, sig.b: The hyperparameters of the priors for the (lognormal) heterogeneity variance, mean and standard deviation respectively.

mu.w, sig.w: The hyperparameters of the priors for the (lognormal) inconsistency variance, mean and standard deviation respectively.

scale: The scaling factor used to create the heavier-tailed distribution from which to sample.

seed: A seed to enable reproduction of results.

The output is a matrix containing one row for each treatment effect and variance component estimate. Each row contains the following: est, the estimate (the posterior mean); sd, the “standard error” (the posterior standard deviation) of each estimate; mcse, the Monte Carlo standard error (of the estimate) and quantiles (2.5%, 50% and 97.5%) of the distributions of the estimates.

The code was run in R version 3.2.1, and each call of 1 million iterations had a run time of approximately 5 minutes (after initial package installation).

*The packages Matrix, mvtnorm, lattice and Hmisc are required for the function to run.*

###################### Function to run many iterations ######################

ismeta <- function(sims, all_data, mu.w, sig.w, mu.b, sig.b, scale=4, seed=1){

set.seed(seed)

####################### Block diagonal S ##############################

require(Matrix) # Necessary for bdiag function

require(mvtnorm) # Necessary for multivariate normal functions

require(lattice) # Required by Hmisc

require(Hmisc) # Necessary for weighted quantile function

# Create and fill matrix S

smat <- bdiag(all_data$s)

smat <- as.matrix(smat)

############## Create and fill matrix X ##############

# Number of treatments in meta-analysis

trts <- 0

for(i in 1:26) trts <- trts + grepl(LETTERS[i], paste(all_data$d,collapse=""))

X <- NULL

# For each study i , create matrix (X1) of dimension

# [number of trts in study i - 1] x [total no. trts in analysis - 1]

for(i in 1:length(all_data$d))

{

des_letters <- strsplit(all_data$d[i], "")[[1]] # Treatments in study

des_numbers <- match(des_letters, LETTERS) # Switch letters for numbers, A=1 etc.

no <- length(des_numbers)-1 # Number of treatments in study - 1

X1=matrix(0, nrow=no, ncol=trts)

for(j in 1:no) X1[j, des_numbers[j+1]] <- 1 # Add 1's in the columns of active treatments;

X1[,des_numbers[1]] <- -1 # Add a -1 in the column of the (relative) reference trt;

X1 <- X1[, -1]

X <- rbind(X, X1, deparse.level = 0) # Combine X1 matrices to create full design matrix X

}

############## Create matrix P1 ##############

p1 <- smat # Same zero terms as S, but

p1[which(p1[,]!=0)] <- 0.5 # all non-zero terms should equal 0.5, except

diag(p1) <- 1 # the diagonal, which should equal 1

############# Create matrix P2 ################

designs <- NULL

for(i in 1:length(all_data$y))

{

new_designs <- rep(all_data$d[i], length(all_data$y[[i]]))

designs <- c(designs, new_designs)

}

Y <- unlist(all_data$y)

n <- length(Y) # Total number of treatment effects

p2 <- matrix(0, nrow=n, ncol=n) # Similar to p1: Entries equal 1 if same design and outcome;

for(i in 1:n) # 0.5 if same design and different outcome; zero otherwise.

for(j in 1:n)

{

if(designs[i]==designs[j] & sum(X[i,]==X[j,])==trts-1) p2[i,j] <- 1

if(designs[i]==designs[j] & sum(X[i,]==X[j,])!=trts-1) p2[i,j] <- 0.5

}

#################### Importance sampling definitions ####################

# Hyperparameters: Lognormal prior variances:

sig2.w <- sig.w^2

sig2.b <- sig.b^2

# Means for lognormal priors:

tau2omega.twid <- exp(mu.w + sig2.w/2)

tau2beta.twid <- exp(mu.b + sig2.b/2)

V.twid <- smat + tau2beta.twid*p1 + tau2omega.twid*p2 # V

V.twid <- as.matrix(V.twid)

var.delta <- solve(t(X) %*% solve(V.twid) %*% X) # Var(delta)

# Estimated average treat. effects:

delta.twid <- solve(t(X) %*% solve(V.twid) %*% X) %*% t(X) %*% solve(V.twid) %*% Y

delta.twid <- as.vector(delta.twid)

tau2beta <- rlnorm(sims, mean=mu.b, sd=sig.b) # Random draws for heterogeneity variance

tau2omega <- rlnorm(sims, mean=mu.w, sd=sig.w) # Random draws for inconsistency variance

# Simulations from heavy-tailed distribution

delta <- rmvnorm(sims, mean=delta.twid, sigma=scale*var.delta)

Ymean <- apply(delta, 1, function(y) X%*%y)

weight_numer <- rep(NA, sims)

# For each iteration:

for(i in 1:sims){

Yvar <- smat + tau2beta[i]*p1 + tau2omega[i]*p2

# Density of desired distribution:

weight_numer[i] <- dmvnorm(Y, mean=Ymean[,i], sigma=Yvar)

}

# Density of simulated data:

weight_sum.wtsom <- apply(delta, 1, dmvnorm, mean=delta.twid, sigma=scale*var.delta)

# Importance sampling weights:

weights <- exp(log(weight_numer)-log(weight_sum.wtsom))

# Weights, treat. effect estimates, variance estimates:

output <- cbind(weights, delta, tau2beta, tau2omega)

all.estimates <- output[,-1]

weighted <- all.estimates*weights

sum.wts <- sum(weights)

# Weighted estimates: sum(estimate*weights)/sum(weights):

est <- colSums(weighted)/sum.wts

sd <- apply(all.estimates, 2, function(x) (sum(weights * x^2/sum(weights)) - (sum(weights * x/sum(weights))^2))^0.5 )

pr <- weights/sum.wts

quants <- apply(all.estimates, 2, wtd.quantile, weights=pr, normwt=TRUE, probs=c(0.025, 0.5, 0.975))

mcse <- apply(all.estimates, 2, function(x) sqrt(sims*var(weights*x)/(sum.wts^2)-2*sum(weights*x)*sims*cov(weights*x, weights)/(sum.wts^3)+((sum(weights*x))^2)*sims*var(weights)/(sum.wts^4)))

result <- cbind(est, sd, mcse, t(quants))

rownames(result) <- c(paste("delta", 2:(dim(result)[1]-1), sep=""), "tau2beta", "tau2omega")

result

}

**Example dataset EG1**

EG1 <- structure(list(y = list(-0.165610916370201, -0.135974059058222,

-0.0801260444792846, -0.147468900468305, 0.093168532086386,

-0.15859403445947, -0.22314355131421, -0.0674412807955327,

-0.118882540989801, -0.0689928714869512, 0.269178600887221,

-0.331609863305099, -0.262364264467491, -0.39319501551635,

-0.115577027634661, 0, -0.40987456407158), s = list(0.0294183340466069,

0.147112449467866, 0.0780588660166125, 0.140361934247383,

0.0479709251030665, 0.0506583523716436, 0.235695187165775,

2.04499494438827, 0.17968120987923, 0.735714285714286, 0.184889643463497,

0.0294022652280727, 0.232478632478632, 0.857874134296899,

0.0219285638496459, 0.168131868131868, 0.0826973577700322),

d = c("CD", "CD", "CE", "CF", "EF", "EF", "EF", "FG", "CH",

"CH", "BC", "AB", "AB", "FG", "AB", "EF", "AE")), .Names = c("y",

"s", "d"))

**Example dataset EG2**

EG2<-structure(list(d = c("AB", "BC", "BC", "BC", "BC", "BC", "BD",

"BD", "CD", "CD", "ABD", "BCD", "BCD"), y = list(-3.61988658,

0, 0.19342045, 2.79320801, 0.24512246, 0.03748309, 0.86020127,

0.14310084, 0.07598591, -0.9903987, c(-1.7408531, 0.3483067

), c(0.4054651, 1.9169226), c(-0.3285041, 1.0732945)), s = list(

0.9672619, 0.4, 0.24987648, 0.61904762, 0.27958937, 0.23845689,

0.04321419, 0.47692308, 0.18416468, 0.61978022, structure(c(0.12650164,

0.07397504, 0.07397504, 0.1583906), .Dim = c(2L, 2L)), structure(c(0.389881,

0.2857143, 0.2857143, 0.5151261), .Dim = c(2L, 2L)), structure(c(0.4361111,

0.2111111, 0.2111111, 0.5380342), .Dim = c(2L, 2L)))), .Names = c("d",

"y", "s"))

**Running the R code**

# EG1 data:

# ismeta(sims=1000000, all_data=EG1, mu.w=-3.5, sig.w=1.26, mu.b=-3.5, sig.b=1.26, scale=4, seed=1)

ismeta(sims=1000000, all_data=EG1, mu.w=-4.803083, sig.w=1.675551, mu.b=-3.5, sig.b=1.26, scale=4, seed=2)

# ismeta(sims=1000000, all_data=EG1, mu.w=-7.992114, sig.w=2.442674, mu.b=-3.5, sig.b=1.26, scale=4, seed=3)

# EG2 data:

# ismeta(sims=1000000, all_data=EG2, mu.w=-2.29, sig.w=1.58, mu.b=-2.29, sig.b=1.58, scale=4, seed=4)

ismeta(sims=1000000, all_data=EG2, mu.w=-3.644406, sig.w=1.954205, mu.b=-2.29, sig.b=1.58, scale=4, seed=5)

# ismeta(sims=1000000, all_data=EG2, mu.w=-6.852633, sig.w=2.648867, mu.b=-2.29, sig.b=1.58, scale=4, seed=6)

**Output**

Dataset EG1

**Inconsistency prior equal to heterogeneity prior**

est sd mcse 2.5% 50.0% 97.5%

delta2 -0.2555 0.2316 0.0018 -0.7354 -0.2516 0.1957

delta3 -0.1645 0.3656 0.0039 -0.8835 -0.1695 0.5594

delta4 -0.3235 0.4614 0.0044 -1.2339 -0.3291 0.5946

delta5 -0.3147 0.3256 0.0034 -0.9518 -0.3084 0.3297

delta6 -0.3510 0.3857 0.0041 -1.1039 -0.3497 0.4133

delta7 -0.6520 0.8998 0.0103 -2.4085 -0.6670 1.1331

delta8 -0.2783 0.5730 0.0058 -1.4007 -0.2783 0.8596

tau2beta 0.0206 0.0225 0.0002 0.0018 0.0136 0.0807

tau2omega 0.0406 0.0629 0.0005 0.0019 0.0218 0.1962

Table 1: R estimates and summaries for dataset EG1, using an inconsistency prior equal to heterogeneity prior.

**Prior mean inconsistency one half of prior mean of heterogeneity (code above)**

est sd mcse 2.5% 50.0% 97.5%

delta2 -0.2463 0.1878 0.0015 -0.6279 -0.2454 0.1136

delta3 -0.1588 0.3375 0.0033 -0.8243 -0.1542 0.5006

delta4 -0.3172 0.4133 0.0038 -1.1319 -0.3174 0.4929

delta5 -0.3105 0.3013 0.0030 -0.8983 -0.3067 0.2745

delta6 -0.3487 0.3456 0.0033 -1.0229 -0.3466 0.3335

delta7 -0.6562 0.8651 0.0085 -2.3397 -0.6693 1.0651

delta8 -0.2729 0.5348 0.0051 -1.3156 -0.2867 0.7957

tau2beta 0.0204 0.0223 0.0002 0.0017 0.0136 0.0792

tau2omega 0.0187 0.0431 0.0002 0.0003 0.0067 0.1112

Table 2: R estimates and summaries for dataset EG1, using an inconsistency prior equal to one half of heterogeneity prior.

**Prior mean inconsistency one tenth of prior mean of heterogeneity**

est sd mcse 2.5% 50.0% 97.5%

delta2 -0.2391 0.1497 0.0011 -0.5348 -0.2383 0.0510

delta3 -0.1655 0.3124 0.0024 -0.7767 -0.1646 0.4515

delta4 -0.3235 0.3720 0.0029 -1.0581 -0.3240 0.4081

delta5 -0.3137 0.2782 0.0022 -0.8617 -0.3154 0.2347

delta6 -0.3528 0.3128 0.0024 -0.9656 -0.3556 0.2647

delta7 -0.6470 0.8462 0.0070 -2.3043 -0.6580 1.0137

delta8 -0.2801 0.5075 0.0040 -1.2804 -0.2727 0.7308

tau2beta 0.0202 0.0218 0.0002 0.0016 0.0136 0.0784

tau2omega 0.0033 0.0176 0.0001 0.0000 0.0003 0.0247

Table 3: R estimates and summaries for dataset EG1, using an inconsistency prior equal to one tenth of heterogeneity prior.

Dataset EG2

**Inconsistency prior equal to heterogeneity prior**

est sd mcse 2.5% 50.0% 97.5%

delta2 -1.8909 0.6560 0.0013 -3.2752 -1.8641 -0.6667

delta3 -1.3338 0.7569 0.0015 -2.9091 -1.3123 0.1034

delta4 -0.6456 0.6905 0.0013 -2.0765 -0.6248 0.6700

tau2beta 0.2230 0.2721 0.0005 0.0055 0.1279 0.9648

tau2omega 0.3516 0.4577 0.0008 0.0085 0.2127 1.5246

Table 4: R estimates and summaries for dataset EG2, using an inconsistency prior equal to heterogeneity prior.

**Prior mean inconsistency one half of prior mean of heterogeneity (code above)**

est sd mcse 2.5% 50.0% 97.5%

delta2 -1.8539 0.6243 0.0012 -3.1765 -1.8249 -0.6907

delta3 -1.3077 0.7065 0.0014 -2.7848 -1.2870 0.0340

delta4 -0.6432 0.6547 0.0013 -2.0119 -0.6242 0.5997

tau2beta 0.2785 0.3064 0.0006 0.0070 0.1810 1.0938

tau2omega 0.2185 0.3801 0.0007 0.0009 0.0817 1.1995

Table 5: R estimates and summaries for dataset EG2, using an inconsistency prior equal to one half of heterogeneity prior.

**Prior mean inconsistency one tenth of prior mean of heterogeneity**

est sd mcse 2.5% 50.0% 97.5%

delta2 -1.8131 0.5938 0.0012 -3.0730 -1.7871 -0.7068

delta3 -1.2794 0.6500 0.0013 -2.6376 -1.2601 -0.0432

delta4 -0.6558 0.6186 0.0012 -1.9600 -0.6335 0.5092

tau2beta 0.3744 0.3400 0.0006 0.0153 0.2867 1.2543

tau2omega 0.0560 0.2054 0.0005 0.0000 0.0017 0.5355

Table 6: R estimates and summaries for dataset EG2, using an inconsistency prior equal to one tenth of heterogeneity prior.
