# Supplementary material for: Two new methods to fit models for network meta-analysis with random inconsistency effects
Source: BMC Med Res Methodol. 2016 Jul 28;16:87. doi: 10.1186/s12874-016-0184-5 (PMC4964019; doi:10.1186/s12874-016-0184-5)
Supplement: Additional file 2 — Matrices M 1 and M 2 from example 2: Topical antibiotics. (PDF 98 kb) [file 12874_2016_184_MOESM2_ESM.pdf]

# Supplementary material: Estimating inconsistency in network meta-analysis using importance sampling

## Matrices $\mathbf{M}_1$ and $\mathbf{M}_2$ from example 2: Topical antibiotics

This dataset comprises thirteen studies with the following study designs: AB, BC, BC, BC, BC, BC, BD, BD, CD, CD, ABD, BCD, BCD. Further details are contained in the Results and Discussion section of the main text.

Description of matrices $\mathbf{M}_1$ and $\mathbf{M}_2$ from Methods section: $\mathbf{M}_1$ contains ones on the main diagonal, so that $\mathbf{M}_{1ii} = 1$ for all $i$. Furthermore, for $i \neq j$, $\mathbf{M}_{1ij} = 1/2$ if the corresponding entries (i.e., rows) of $\mathbf{Y}$ are from the same study, and $\mathbf{M}_{1ij} = 0$ otherwise. We similarly define $\mathbf{M}_2$ as containing ones on the main diagonal, so that $\mathbf{M}_{2ii} = 1$ for all $i$. Furthermore, for $i \neq j$, $\mathbf{M}_{2ij} = 1$ if the corresponding entries of $\mathbf{Y}$ are from the same design and refer to the same treatment effect, $\mathbf{M}_{2ij} = 1/2$ if they are from the same design but refer to different treatment effects, and $\mathbf{M}_{2ij} = 0$ otherwise. Below, we have added horziontal and vertical lines to demarcate different studies in $\mathbf{M}_1$ and different designs in $\mathbf{M}_2$.

$$
\mathbf{M_1} = \left(\begin{array}{c|c|c|c|c|c|c|c|c|c|cc|cc|cc}
1 & 0 & 0 & 0 & 0 & 0 & 0 & 0 & 0 & 0 & 0 & 0 & 0 & 0 & 0 & 0 \\ \hline
0 & 1 & 0 & 0 & 0 & 0 & 0 & 0 & 0 & 0 & 0 & 0 & 0 & 0 & 0 & 0 \\ \hline
0 & 0 & 1 & 0 & 0 & 0 & 0 & 0 & 0 & 0 & 0 & 0 & 0 & 0 & 0 & 0 \\ \hline
0 & 0 & 0 & 1 & 0 & 0 & 0 & 0 & 0 & 0 & 0 & 0 & 0 & 0 & 0 & 0 \\ \hline
0 & 0 & 0 & 0 & 1 & 0 & 0 & 0 & 0 & 0 & 0 & 0 & 0 & 0 & 0 & 0 \\ \hline
0 & 0 & 0 & 0 & 0 & 1 & 0 & 0 & 0 & 0 & 0 & 0 & 0 & 0 & 0 & 0 \\ \hline
0 & 0 & 0 & 0 & 0 & 0 & 1 & 0 & 0 & 0 & 0 & 0 & 0 & 0 & 0 & 0 \\ \hline
0 & 0 & 0 & 0 & 0 & 0 & 0 & 1 & 0 & 0 & 0 & 0 & 0 & 0 & 0 & 0 \\ \hline
0 & 0 & 0 & 0 & 0 & 0 & 0 & 0 & 1 & 0 & 0 & 0 & 0 & 0 & 0 & 0 \\ \hline
0 & 0 & 0 & 0 & 0 & 0 & 0 & 0 & 0 & 1 & 0 & 0 & 0 & 0 & 0 & 0 \\ \hline
0 & 0 & 0 & 0 & 0 & 0 & 0 & 0 & 0 & 0 & 1 & \frac{1}{2} & 0 & 0 & 0 & 0 \\
0 & 0 & 0 & 0 & 0 & 0 & 0 & 0 & 0 & 0 & \frac{1}{2} & 1 & 0 & 0 & 0 & 0 \\ \hline
0 & 0 & 0 & 0 & 0 & 0 & 0 & 0 & 0 & 0 & 0 & 0 & 1 & \frac{1}{2} & 0 & 0 \\
0 & 0 & 0 & 0 & 0 & 0 & 0 & 0 & 0 & 0 & 0 & 0 & \frac{1}{2} & 1 & 0 & 0 \\ \hline
0 & 0 & 0 & 0 & 0 & 0 & 0 & 0 & 0 & 0 & 0 & 0 & 0 & 0 & 1 & \frac{1}{2} \\
0 & 0 & 0 & 0 & 0 & 0 & 0 & 0 & 0 & 0 & 0 & 0 & 0 & 0 & \frac{1}{2} & 1
\end{array}\right)
$$

$$
\mathbf{M_2} = \left(\begin{array}{c|ccccc|cc|cc|cc|cccc}
1 & 0 & 0 & 0 & 0 & 0 & 0 & 0 & 0 & 0 & 0 & 0 & 0 & 0 & 0 & 0 \\ \hline
0 & 1 & 1 & 1 & 1 & 1 & 0 & 0 & 0 & 0 & 0 & 0 & 0 & 0 & 0 & 0 \\
0 & 1 & 1 & 1 & 1 & 1 & 0 & 0 & 0 & 0 & 0 & 0 & 0 & 0 & 0 & 0 \\
0 & 1 & 1 & 1 & 1 & 1 & 0 & 0 & 0 & 0 & 0 & 0 & 0 & 0 & 0 & 0 \\
0 & 1 & 1 & 1 & 1 & 1 & 0 & 0 & 0 & 0 & 0 & 0 & 0 & 0 & 0 & 0 \\
0 & 1 & 1 & 1 & 1 & 1 & 0 & 0 & 0 & 0 & 0 & 0 & 0 & 0 & 0 & 0 \\ \hline
0 & 0 & 0 & 0 & 0 & 0 & 1 & 1 & 0 & 0 & 0 & 0 & 0 & 0 & 0 & 0 \\
0 & 0 & 0 & 0 & 0 & 0 & 1 & 1 & 0 & 0 & 0 & 0 & 0 & 0 & 0 & 0 \\ \hline
0 & 0 & 0 & 0 & 0 & 0 & 0 & 0 & 1 & 1 & 0 & 0 & 0 & 0 & 0 & 0 \\
0 & 0 & 0 & 0 & 0 & 0 & 0 & 0 & 1 & 1 & 0 & 0 & 0 & 0 & 0 & 0 \\ \hline
0 & 0 & 0 & 0 & 0 & 0 & 0 & 0 & 0 & 0 & 1 & \frac{1}{2} & 0 & 0 & 0 & 0 \\
0 & 0 & 0 & 0 & 0 & 0 & 0 & 0 & 0 & 0 & \frac{1}{2} & 1 & 0 & 0 & 0 & 0 \\ \hline
0 & 0 & 0 & 0 & 0 & 0 & 0 & 0 & 0 & 0 & 0 & 0 & 1 & \frac{1}{2} & 1 & \frac{1}{2} \\
0 & 0 & 0 & 0 & 0 & 0 & 0 & 0 & 0 & 0 & 0 & 0 & \frac{1}{2} & 1 & \frac{1}{2} & 1 \\
0 & 0 & 0 & 0 & 0 & 0 & 0 & 0 & 0 & 0 & 0 & 0 & 1 & \frac{1}{2} & 1 & \frac{1}{2} \\
0 & 0 & 0 & 0 & 0 & 0 & 0 & 0 & 0 & 0 & 0 & 0 & \frac{1}{2} & 1 & \frac{1}{2} & 1
\end{array}\right)
$$
