# Supplementary material for: Two new methods to fit models for network meta-analysis with random inconsistency effects
Source: BMC Med Res Methodol. 2016 Jul 28;16:87. doi: 10.1186/s12874-016-0184-5 (PMC4964019; doi:10.1186/s12874-016-0184-5)
Supplement: Additional file 3 — Difference between R and BUGS results. (DOCX 5 kb) [file 12874_2016_184_MOESM3_ESM.docx]

**Supplementary material: Estimating inconsistency in network meta-analysis using importance sampling**

**Difference between R and BUGS results**

Dataset EG1

|  | **Difference between R and BUGS output (SE)** | | |
| --- | --- | --- | --- |
|  | **Inconsistency prior equal to heterogeneity prior** | **Prior mean inconsistency one half of prior mean of heterogeneity** | **Prior mean inconsistency one tenth of prior mean of heterogeneity** |
| delta2 | 0.0016 (0.0025) | 0.0008 (0.0019) | -0.0001 (0.0012) |
| delta3 | 0.0035 (0.0057) | -0.0041 (0.0048) | 0.0034 (0.0038) |
| delta4 | 0.0054 (0.0064) | -0.0047 (0.0055) | 0.0022 (0.0042) |
| delta5 | 0.0015 (0.0050) | -0.0015 (0.0043) | 0.0021 (0.0034) |
| delta6 | 0.0033 (0.0060) | 0.0006 (0.0049) | 0.0024 (0.0038) |
| delta7 | 0.0065 (0.0148) | 0.0214 (0.0132) | 0.0024 (0.0119) |
| delta8 | 0.0094 (0.0080) | 0.0008 (0.0070) | 0.0081 (0.0058) |
| tau2beta | 0.0000 (0.0002) | 0.0001 (0.0002) | 0.0000 (0.0002) |
| tau2omega | 0.0007 (0.0007) | -0.0003 (0.0004) | -0.0003 (0.0001) |

Table 7: EG1: Difference between R and BUGS estimates (SE: Square root of sum of squared MC errors)

Dataset EG2

|  | **Difference between R and BUGS output (SE)** | | |
| --- | --- | --- | --- |
|  | **Inconsistency prior equal to heterogeneity prior** | **Prior mean inconsistency one half of prior mean of heterogeneity** | **Prior mean inconsistency one tenth of prior mean of heterogeneity** |
| delta2 | -0.0041 (0.0065) | 0.0059 (0.0053) | -0.0019 (0.0040) |
| delta3 | -0.0042 (0.0075) | 0.0067 (0.0062) | -0.0056 (0.0044) |
| delta4 | -0.0039 (0.0065) | 0.0054 (0.0055) | -0.0032 (0.0040) |
| tau2beta | 0.0002 (0.0015) | 0.0016 (0.0018) | 0.0005 (0.0018) |
| tau2omega | -0.0036 (0.0032) | -0.0023 (0.0031) | 0.0027 (0.0023) |

Table 8: EG2: Difference in Monte Carlo error between R and BUGS estimates (SE: Square root of sum of squared MC errors)
