# Supplementary material for: Two new methods to fit models for network meta-analysis with random inconsistency effects
Source: BMC Med Res Methodol. 2016 Jul 28;16:87. doi: 10.1186/s12874-016-0184-5 (PMC4964019; doi:10.1186/s12874-016-0184-5)
Supplement: Additional file 4 — OpenBUGS code. (DOCX 41 kb) [file 12874_2016_184_MOESM4_ESM.docx]

**Supplementary material: Estimating inconsistency in network meta-analysis using importance sampling**

**OpenBUGS code**

The BUGS models below estimate treatment effects and variance components for our two network meta-analysis examples. This code has been adapted from the code given in the supplementary materials of Jackson et al [cite design-by-treatment interaction model]. An important distinction between the R and BUGS codes is that while the supplied R code can accommodate any network (up to a maximum of 26 treatments), the BUGS code must be altered depending on the greatest number of arms contained in any single study within the meta-analysis. This is because the model must account for the dimension of the largest within-study variance-covariance matrix. We include two example datasets, one in which there are only 2-arm trials (EG1) and one in which there is a combination of 2- and 3-arm trials (EG2). Thus, we include two BUGS models.

In each example, the trial data are loaded as a combination of matrices and vectors with number of rows (or length, in the case of vectors) equal to the number of studies. In example EG2, these are as follows:

t: Matrix of numbers representing the treatments in each study (A=1, B=2, etc.);

y: Matrix of estimated treatment effects;

var: Matrix of within-study variances;

cov: Vector of within-study covariances.

In this example, the first column of t, t[i,1], contains the reference treatment; the second column, t[i,2], contains the non-reference treatment for the 2-arm studies and the first non-reference treatment for the 3-arm studies; the third column, t[i,3], contains NA values for the 2-arm studies and the second non-reference treatment of the 3-arm studies. Similarly, the first column of y, y[i,1], contains the estimated treatment effects for the 2-arm studies and the first contrast of the 3-arm studies, while the second column of y, y[i,2], contains NA values for the 2-arm studies and the estimated treatment effect of the second contrast of the 3-arm studies. The matrix var and the vector cov contain the within-study variances and covariances respectively in the same way. Note that for a network containing a 4-arm (or greater) study, cov is required to be a matrix.

Other data required in addition to the model code are the following:

nt: Total number of treatments across all studies (including reference treatment);

ns2: Number of 2-arm studies;

ns3: Number of 3-arm studies;

ndes: Number of unique designs

nades: Number of arms in each unique design

The initial values used are:

tau2beta: Heterogeneity variance;

tau2omega: Inconsistency variance;

delta: Treatment effect estimates (first entry – reference treatment – should be NA).

Note: The hyperparameters on the priors must be expressed within the model; this is in contrast to the R code, where the hyperparameters are specified outside the code as function arguments.

The models were compiled in OpenBUGS version 3.2.3 for 1 million iterations and a burn-in of 100,000. The runtime was approximately 3 minutes for each dataset.

In the first example, all studies are two arm studies. Hence some of the data provided are not needed in this example (eg cov) which results in OpenBUGS providing a warning message. However, to keep the data structure the same for both examples, data of this type are given for both examples.

**Full BUGS model for a network containing 2-arm studies only (e.g. example dataset EG1):**

model{

# NORMAL LIKELIHOOD

# LOOP THROUGH 2-ARM STUDIES

for(i in 1:ns2){

mu[i,2] <- eta[i,2] + om[des[i],2]

prec[i] <- 1/var[i,2]

y[i,2] ~ dnorm(mu[i,2],prec[i])

}

# RANDOM EFFECTS DISTRIBUTION

for(i in 1:(ns2+ns3)){ # LOOP THROUGH ALL STUDIES

w[i,1] <- 0

eta[i,1] <- 0

for(k in 2:na[i]){ # LOOP THROUGH ARMS

eta[i,k] ~ dnorm(m.cond[i,k],precbeta.cond[i,k])

# MEANS WITH MULTI-ARM TRIAL CORRECTION

m.cond[i,k] <- delta[t[i,k]] - delta[t[i,1]] + sw[i,k]

# BETWEEN-STUDY PRECISION WITH MULTI-ARM TRIAL CORRECTION

precbeta.cond[i,k] <- precbeta * 2 * (k-1) /k

w[i,k] <- (eta[i,k] - delta[t[i,k]] + delta[t[i,1]])

sw[i,k] <- sum(w[i,1:(k-1)])/(k-1)

}

}

# INCONSISTENCY PARAMETERS

for (i in 1:ndes) { # LOOP THROUGH DESIGNS

om[i,1] <- 0

for(k in 2:nades[i]) { # LOOP THROUGH ARMS OF DESIGN i

om[i,k] ~ dnorm(mom.cond[i,k],precom.cond[i,k])

# MEAN OF INCONSISTENCY DISTRIBUTION WITH MULTI-ARM TRIAL CORRECTION

mom.cond[i,k] <- sum(om[i,1:k-1])/(k-1)

# PRECISION OF INCONSISTENCY DISTRIBUTION WITH MULTI-ARM TRIAL CORRECTION

precom.cond[i,k] <- precomega * 2 * (k-1)/k

}

}

# TREATMENT EFFECT IS ZERO FOR REFERENCE TREATMENT

delta[1]<-0

# PRIORS

for (k in 2:nt){ delta[k] ~ dunif(-10, 10) }

tau2beta ~ dlnorm(mu.b,prior.prec.beta)

prior.prec.beta<-1/(sig.b*sig.b)

precbeta <- pow(tau2beta,-1)

tau2omega ~ dlnorm(mu.w,prior.prec.omega)

prior.prec.omega<-1/(sig.w*sig.w)

precomega <- pow(tau2omega,-1)

# Hyperparameters for EG1 dataset:

mu.b <- -3.5

sig.b <- 1.26

mu.w <- -4.803083

sig.w <- 1.675551

}

**Inconsistency priors for dataset EG1**

Inconsistency prior equal to heterogeneity prior:

mu.w <- -3.5

sig.w <- 1.26

Prior mean inconsistency one half of prior mean of heterogeneity (used in code above):

mu.w <- -4.803083

sig.w <- 1.675551

Prior mean inconsistency one tenth of prior mean of heterogeneity:

mu.w <- -7.992114

sig.w <- 2.442674

**Example dataset EG1**

# Details of network:

list(nt=8,ns2=17,ns3=0,ndes=9,nades= c(2, 2, 2, 2, 2, 2, 2, 2, 2))

# Dataset:

t[,1] t[,2] y[,2] var[,2] t[,3] y[,3] var[,3] t[,4] y[,4] var[,4] cov[] na[] no[] des[]

3 4 -0.16561092 0.02941833 NA NA NA NA NA NA NA 2 1 1

3 4 -0.13597406 0.14711245 NA NA NA NA NA NA NA 2 1 1

3 5 -0.08012604 0.07805887 NA NA NA NA NA NA NA 2 1 2

3 6 -0.14746890 0.14036193 NA NA NA NA NA NA NA 2 1 3

5 6 0.09316853 0.04797093 NA NA NA NA NA NA NA 2 1 4

5 6 -0.15859403 0.05065835 NA NA NA NA NA NA NA 2 1 4

5 6 -0.22314355 0.23569519 NA NA NA NA NA NA NA 2 1 4

6 7 -0.06744128 2.04499494 NA NA NA NA NA NA NA 2 1 5

3 8 -0.11888254 0.17968121 NA NA NA NA NA NA NA 2 1 6

3 8 -0.06899287 0.73571429 NA NA NA NA NA NA NA 2 1 6

2 3 0.26917860 0.18488964 NA NA NA NA NA NA NA 2 1 7

1 2 -0.33160986 0.02940227 NA NA NA NA NA NA NA 2 1 8

1 2 -0.26236426 0.23247863 NA NA NA NA NA NA NA 2 1 8

6 7 -0.39319502 0.85787413 NA NA NA NA NA NA NA 2 1 5

1 2 -0.11557703 0.02192856 NA NA NA NA NA NA NA 2 1 8

5 6 0.00000000 0.16813187 NA NA NA NA NA NA NA 2 1 4

1 5 -0.40987456 0.08269736 NA NA NA NA NA NA NA 2 1 9

END

**Initial values for dataset EG1**

list(tau2beta=0.01, tau2omega=0.01 )

list(delta=c(NA,0,0,0,0,0,0,0))

**Full BUGS model for a network containing 2- and 3-arm studies (e.g. example dataset EG2)**

model{

# LOOP THROUGH 2-ARM STUDIES

for(i in 1:ns2){

mu[i,2] <- eta[i,2] + om[des[i],2]

prec[i] <- 1/var[i,2]

y[i,2] ~ dnorm(mu[i,2],prec[i])

}

# LOOP THROUGH 3-ARM STUDIES

for(i in (ns2+1):(ns2+ns3)){

for(k in 1:no[i]){

for(j in 1:no[i]){

# WITHIN-STUDY COVARIANCE

Sigma[i,j,k] <- cov[i]*(1-equals(j,k))+var[i,k+1]*equals(j,k)

}

# TRUE TREATMENT EFFECT IN STUDY i

mu[i,k+1] <- eta[i,k+1]+om[des[i],k+1]

}

# WITHIN-STUDY PRECISION

Prec[i,1:no[i],1:no[i]] <- inverse(Sigma[i,,])

y[i,2:(no[i]+1)] ~ dmnorm(mu[i,2:(no[i]+1)],Prec[i,1:no[i],1:no[i]])

}

# RANDOM EFFECTS DISTRIBUTION

for(i in 1:(ns2+ns3)){ # LOOP THROUGH ALL STUDIES

w[i,1] <- 0

eta[i,1] <- 0

for(k in 2:na[i]){ # LOOP THROUGH ARMS

eta[i,k] ~ dnorm(m.cond[i,k],precbeta.cond[i,k])

# MEANS WITH MULTI-ARM TRIAL CORRECTION

m.cond[i,k] <- delta[t[i,k]] - delta[t[i,1]] + sw[i,k]

# BETWEEN-STUDY PRECISION WITH MULTI-ARM TRIAL CORRECTION

precbeta.cond[i,k] <- precbeta * 2 * (k-1) /k

w[i,k] <- (eta[i,k] - delta[t[i,k]] + delta[t[i,1]])

sw[i,k] <- sum(w[i,1:(k-1)])/(k-1)

}

}

# INCONSISTENCY PARAMETERS

for (i in 1:ndes) { # LOOP THROUGH DESIGNS

om[i,1] <- 0

for(k in 2:nades[i]) { # LOOP THROUGH ARMS OF DESIGN i

om[i,k] ~ dnorm(mom.cond[i,k],precom.cond[i,k])

# MEAN OF INCONSISTENCY DISTRIBUTION WITH MULTI-ARM TRIAL CORRECTION

mom.cond[i,k] <- sum(om[i,1:k-1])/(k-1)

# PRECISION OF INCONSISTENCY DISTRIBUTION WITH MULTI-ARM TRIAL CORRECTION

precom.cond[i,k] <- precomega * 2 * (k-1)/k

}

}

# TREATMENT EFFECT IS ZERO FOR REFERENCE TREATMENT

delta[1]<-0

# PRIORS

for (k in 2:nt){ delta[k] ~ dunif(-10, 10) }

tau2beta ~ dlnorm(mu.b,prior.prec.beta)

prior.prec.beta<-1/(sig.b*sig.b)

precbeta <- pow(tau2beta,-1)

tau2omega ~ dlnorm(mu.w,prior.prec.omega)

prior.prec.omega<- 1/(sig.w*sig.w)

precomega <- pow(tau2omega,-1)

# Hyperparameters for EG2 dataset:

mu.b <- -2.29

sig.b <- 1.58

mu.w <- -3.644406

sig.w <- 1.954205

}

**Inconsistency priors for dataset EG2**

Inconsistency prior equal to heterogeneity prior:

mu.w <- -2.29

sig.w <- 1.58

Prior mean inconsistency one half of prior mean of heterogeneity (used in code above):

mu.w <- -3.644406

sig.w <- 1.954205

Prior mean inconsistency one tenth of prior mean of heterogeneity:

mu.w <- -6.852633

sig.w <- 2.648867

**Example dataset EG2**

# Details of network:

list(nt=4,ns2=10,ns3=3,ndes=6,nades= c(2, 2, 2, 2, 3, 3 ))

# Dataset:

t[,1]t[,2] y[,2] var[,2] t[,3] y[,3] var[,3] t[,4] y[,4] var[,4] cov[] na[] no[] des[]

1 2 -3.61988658 0.96726190 NA NA NA NA NA NA NA 2 1 1

2 3 0.00000000 0.40000000 NA NA NA NA NA NA NA 2 1 2

2 3 0.19342045 0.24987648 NA NA NA NA NA NA NA 2 1 2

2 3 2.79320801 0.61904762 NA NA NA NA NA NA NA 2 1 2

2 3 0.24512246 0.27958937 NA NA NA NA NA NA NA 2 1 2

2 3 0.03748309 0.23845689 NA NA NA NA NA NA NA 2 1 2

2 4 0.86020127 0.04321419 NA NA NA NA NA NA NA 2 1 3

2 4 0.14310084 0.47692308 NA NA NA NA NA NA NA 2 1 3

3 4 0.07598591 0.18416468 NA NA NA NA NA NA NA 2 1 4

3 4 -0.99039870 0.61978022 NA NA NA NA NA NA NA 2 1 4

1 2 -1.7408531 0.12650164 4 0.3483067 0.15839063 NA NA NA 0.07397504 3 2 5

2 3 0.4054651 0.3898810 4 1.9169226 0.5151261 NA NA NA 0.2857143 3 2 6

2 3 -0.3285041 0.4361111 4 1.0732945 0.5380342 NA NA NA 0.2111111 3 2 6

END

**Initial values for dataset EG2**

list(tau2beta=0.01, tau2omega=0.01 )

list(delta=c(NA, 0,0,0))

**Output**

Dataset EG1

**Inconsistency prior equal to heterogeneity prior**

|  | **mean** | **sd** | **MC_error** | **val2.5pc** | **median** | **val97.5pc** | **start** | **sample** |
| --- | --- | --- | --- | --- | --- | --- | --- | --- |
| delta[2] | -0.2539 | 0.2341 | 0.001663 | -0.7322 | -0.2498 | 0.1995 | 100001 | 1000000 |
| delta[3] | -0.161 | 0.3647 | 0.004152 | -0.878 | -0.1619 | 0.5596 | 100001 | 1000000 |
| delta[4] | -0.3181 | 0.4588 | 0.004597 | -1.214 | -0.32 | 0.5911 | 100001 | 1000000 |
| delta[5] | -0.3132 | 0.3278 | 0.003598 | -0.9564 | -0.3141 | 0.3313 | 100001 | 1000000 |
| delta[6] | -0.3477 | 0.3839 | 0.004432 | -1.109 | -0.3485 | 0.4091 | 100001 | 1000000 |
| delta[7] | -0.6455 | 0.8953 | 0.01063 | -2.408 | -0.64 | 1.105 | 100001 | 1000000 |
| delta[8] | -0.2689 | 0.5744 | 0.005513 | -1.388 | -0.2707 | 0.8627 | 100001 | 1000000 |
| tau2beta | 0.02058 | 0.02257 | 8.74E-5 | 0.001689 | 0.01366 | 0.08029 | 100001 | 1000000 |
| tau2omega | 0.04131 | 0.06428 | 5.305E-4 | 0.002204 | 0.02216 | 0.1966 | 100001 | 1000000 |

Table 1: OpenBUGS estimates and summaries for dataset EG1, using an inconsistency prior equal to heterogeneity prior.

**Prior mean inconsistency one half of prior mean of heterogeneity (code above)**

| \|  \|  \| **mean** \| **sd** \| **MC_error** \| **val2.5pc** \| **median** \| **val97.5pc** \| **start** \| **sample** \| \| --- \| --- \| --- \| --- \| --- \| --- \| --- \| --- \| --- \| --- \| \|  \| delta[2] \| -0.2455 \| 0.1887 \| 0.001129 \| -0.6258 \| -0.2428 \| 0.1166 \| 100001 \| 1000000 \| \|  \| delta[3] \| -0.1629 \| 0.3412 \| 0.003585 \| -0.8303 \| -0.1649 \| 0.5098 \| 100001 \| 1000000 \| \|  \| delta[4] \| -0.3219 \| 0.4168 \| 0.003927 \| -1.137 \| -0.3234 \| 0.4998 \| 100001 \| 1000000 \| \|  \| delta[5] \| -0.312 \| 0.3007 \| 0.003038 \| -0.9047 \| -0.3128 \| 0.2836 \| 100001 \| 1000000 \| \|  \| delta[6] \| -0.3481 \| 0.3463 \| 0.003633 \| -1.029 \| -0.3481 \| 0.3375 \| 100001 \| 1000000 \| \|  \| delta[7] \| -0.6348 \| 0.8639 \| 0.01004 \| -2.334 \| -0.635 \| 1.064 \| 100001 \| 1000000 \| \|  \| delta[8] \| -0.2721 \| 0.5381 \| 0.00479 \| -1.323 \| -0.2739 \| 0.7838 \| 100001 \| 1000000 \| \|  \| tau2beta \| 0.02045 \| 0.02237 \| 8.107E-5 \| 0.001694 \| 0.01366 \| 0.07934 \| 100001 \| 1000000 \| \|  \| tau2omega \| 0.01839 \| 0.04412 \| 3.229E-4 \| 2.785E-4 \| 0.006415 \| 0.1119 \| 100001 \| 1000000 \| |
| --- | --- | --- | --- | --- | --- | --- | --- | --- | --- | --- | --- | --- | --- | --- | --- | --- | --- | --- | --- | --- | --- | --- | --- | --- | --- | --- | --- | --- | --- | --- | --- | --- | --- | --- | --- | --- | --- | --- | --- | --- | --- | --- | --- | --- | --- | --- | --- | --- | --- | --- | --- | --- | --- | --- | --- | --- | --- | --- | --- | --- | --- | --- | --- | --- | --- | --- | --- | --- | --- | --- | --- | --- | --- | --- | --- | --- | --- | --- | --- | --- | --- | --- | --- | --- | --- | --- | --- | --- | --- | --- | --- | --- | --- | --- | --- | --- | --- | --- | --- | --- |

Table 2: OpenBUGS estimates and summaries for dataset EG1, using an inconsistency prior equal to one half of heterogeneity prior.

**Prior mean inconsistency one tenth of prior mean of heterogeneity**

|  | **mean** | **sd** | **MC_error** | **val2.5pc** | **median** | **val97.5pc** | **start** | **sample** |
| --- | --- | --- | --- | --- | --- | --- | --- | --- |
| delta[2] | -0.2392 | 0.1486 | 5.785E-4 | -0.5366 | -0.2376 | 0.04901 | 100001 | 1000000 |
| delta[3] | -0.1621 | 0.3124 | 0.00295 | -0.7796 | -0.1619 | 0.4472 | 100001 | 1000000 |
| delta[4] | -0.3213 | 0.3728 | 0.003136 | -1.055 | -0.3207 | 0.4115 | 100001 | 1000000 |
| delta[5] | -0.3116 | 0.2782 | 0.002561 | -0.8597 | -0.3108 | 0.2324 | 100001 | 1000000 |
| delta[6] | -0.3504 | 0.3121 | 0.002941 | -0.9644 | -0.3495 | 0.2627 | 100001 | 1000000 |
| delta[7] | -0.6446 | 0.8364 | 0.009598 | -2.274 | -0.6423 | 1.003 | 100001 | 1000000 |
| delta[8] | -0.272 | 0.5056 | 0.004152 | -1.271 | -0.2711 | 0.7162 | 100001 | 1000000 |
| tau2beta | 0.02012 | 0.02188 | 8.33E-5 | 0.001691 | 0.01349 | 0.07805 | 100001 | 1000000 |
| tau2omega | 0.003055 | 0.01309 | 8.328E-5 | 2.641E-6 | 3.12E-4 | 0.02416 | 100001 | 1000000 |

Table 3: OpenBUGS estimates and summaries for dataset EG1, using an inconsistency prior equal to one tenth of heterogeneity prior.

Dataset EG2

**Inconsistency prior equal to heterogeneity prior**

|  | **mean** | **sd** | **MC_error** | **val2.5pc** | **median** | **val97.5pc** | **start** | **sample** |
| --- | --- | --- | --- | --- | --- | --- | --- | --- |
| delta[2] | -1.895 | 0.6584 | 0.006369 | -3.295 | -1.864 | -0.6658 | 100001 | 1000000 |
| delta[3] | -1.338 | 0.7559 | 0.00732 | -2.918 | -1.312 | 0.09029 | 100001 | 1000000 |
| delta[4] | -0.6495 | 0.6897 | 0.006402 | -2.097 | -0.6241 | 0.6542 | 100001 | 1000000 |
| tau2beta | 0.2232 | 0.2733 | 0.001412 | 0.005535 | 0.1277 | 0.9667 | 100001 | 1000000 |
| tau2omega | 0.348 | 0.4508 | 0.003135 | 0.008257 | 0.2088 | 1.511 | 100001 | 1000000 |

Table 4: OpenBUGS estimates and summaries for dataset EG2, using an inconsistency prior equal to heterogeneity prior.

**Prior mean inconsistency one half of prior mean of heterogeneity (code above)**

|  | **mean** | **sd** | **MC_error** | **val2.5pc** | **median** | **val97.5pc** | **start** | **sample** |
| --- | --- | --- | --- | --- | --- | --- | --- | --- |
| delta[2] | -1.848 | 0.6212 | 0.005186 | -3.148 | -1.823 | -0.6836 | 100001 | 1000000 |
| delta[3] | -1.301 | 0.7074 | 0.006054 | -2.766 | -1.281 | 0.04299 | 100001 | 1000000 |
| delta[4] | -0.6378 | 0.6533 | 0.005348 | -1.986 | -0.6203 | 0.6085 | 100001 | 1000000 |
| tau2beta | 0.2801 | 0.3066 | 0.00168 | 0.007226 | 0.1834 | 1.093 | 100001 | 1000000 |
| tau2omega | 0.2162 | 0.3759 | 0.003016 | 9.474E-4 | 0.0806 | 1.19 | 100001 | 1000000 |

Table 5: OpenBUGS estimates and summaries for dataset EG2, using an inconsistency prior equal to one half of heterogeneity prior.

**Prior mean inconsistency one tenth of prior mean of heterogeneity**

|  | **mean** | **sd** | **MC_error** | **val2.5pc** | **median** | **val97.5pc** | **start** | **sample** |
| --- | --- | --- | --- | --- | --- | --- | --- | --- |
| delta[2] | -1.815 | 0.5972 | 0.003851 | -3.08 | -1.786 | -0.7041 | 100001 | 1000000 |
| delta[3] | -1.285 | 0.6562 | 0.004244 | -2.647 | -1.262 | -0.04086 | 100001 | 1000000 |
| delta[4] | -0.659 | 0.6232 | 0.003821 | -1.973 | -0.6348 | 0.5139 | 100001 | 1000000 |
| tau2beta | 0.3749 | 0.341 | 0.001674 | 0.01506 | 0.2868 | 1.258 | 100001 | 1000000 |
| tau2omega | 0.05872 | 0.2264 | 0.002282 | 6.727E-6 | 0.001695 | 0.5653 | 100001 | 1000000 |

Table 6: OpenBUGS estimates and summaries for dataset EG2, using an inconsistency prior equal to one tenth of heterogeneity prior.
