# Supplementary material for: Two new methods to fit models for network meta-analysis with random inconsistency effects
Source: BMC Med Res Methodol. 2016 Jul 28;16:87. doi: 10.1186/s12874-016-0184-5 (PMC4964019; doi:10.1186/s12874-016-0184-5)
Supplement: Additional file 5 — Fitting Jackson’s model using metafor. (DOCX 31 kb) [file 12874_2016_184_MOESM5_ESM.docx]

**Supplementary material: Fitting Jackson’s model using metafor**

**R code**

The code below allows the user to fit the consistency model and Jackson’s model in R using the *metafor* package. The data for each example have been arranged in the format required for the analyses. Each dataset (EG1 and EG2) includes the following variables:

- study: study id numbers
- y: the estimated treatment effects
- ref: the reference treatment for a particular study
- trt: the treatment against which ‘ref’ is compared
- contr: the contrast (‘ref’ and ‘trt’ concatenated)
- design: the design of the study

In addition, S1 and S2 are the corresponding within-study covariance matrices of the errors ε.

The following convenience function will be used below to create the design matrix X for a particular dataset.

contrmat <- function(trt1, trt2, ref) {

all.lvls <- sort(unique(c(levels(factor(trt1)), levels(factor(trt2)))))

trt1 <- factor(trt1, levels=all.lvls)

trt2 <- factor(trt2, levels=all.lvls)

X <- model.matrix(~ trt2 - 1) - model.matrix(~ trt1 - 1)

colnames(X) <- all.lvls

if (missing(ref))

ref <- all.lvls[1]

X[, colnames(X) != ref]

}

Package *metafor* (at least version 1.9.8) is required for the analyses. The package can be installed and loaded with:

install.packages("metafor")

library(metafor)

Note that variance components $\tau_{\beta}^{2}$ and $\tau_{\omega}^{2}$ as described in the paper are denoted as tau^2 and gamma^2 in the output below.

**Example dataset EG1**

EG1 <- read.table(header=TRUE, as.is=TRUE, text="

study y ref trt contr design

1 -0.16561092 C D CD CD

2 -0.13597406 C D CD CD

3 -0.08012604 C E CE CE

4 -0.14746890 C F CF CF

5 0.09316853 E F EF EF

6 -0.15859403 E F EF EF

7 -0.22314355 E F EF EF

8 -0.06744128 F G FG FG

9 -0.11888254 C H CH CH

10 -0.06899287 C H CH CH

11 0.26917860 B C BC BC

12 -0.33160986 A B AB AB

13 -0.26236426 A B AB AB

14 -0.39319502 F G FG FG

15 -0.11557703 A B AB AB

16 0.00000000 E F EF EF

17 -0.40987456 A E AE AE

")

S1 <- structure(c(0.0294183340466069, 0, 0, 0, 0, 0, 0, 0, 0, 0, 0,

0, 0, 0, 0, 0, 0, 0, 0.147112449467866, 0, 0, 0, 0, 0, 0, 0,

0, 0, 0, 0, 0, 0, 0, 0, 0, 0, 0.0780588660166125, 0, 0, 0, 0,

0, 0, 0, 0, 0, 0, 0, 0, 0, 0, 0, 0, 0, 0.140361934247383, 0,

0, 0, 0, 0, 0, 0, 0, 0, 0, 0, 0, 0, 0, 0, 0, 0, 0.0479709251030665,

0, 0, 0, 0, 0, 0, 0, 0, 0, 0, 0, 0, 0, 0, 0, 0, 0, 0.0506583523716436,

0, 0, 0, 0, 0, 0, 0, 0, 0, 0, 0, 0, 0, 0, 0, 0, 0, 0.235695187165775,

0, 0, 0, 0, 0, 0, 0, 0, 0, 0, 0, 0, 0, 0, 0, 0, 0, 2.04499494438827,

0, 0, 0, 0, 0, 0, 0, 0, 0, 0, 0, 0, 0, 0, 0, 0, 0, 0.17968120987923,

0, 0, 0, 0, 0, 0, 0, 0, 0, 0, 0, 0, 0, 0, 0, 0, 0, 0.735714285714286,

0, 0, 0, 0, 0, 0, 0, 0, 0, 0, 0, 0, 0, 0, 0, 0, 0, 0.184889643463497,

0, 0, 0, 0, 0, 0, 0, 0, 0, 0, 0, 0, 0, 0, 0, 0, 0, 0.0294022652280727,

0, 0, 0, 0, 0, 0, 0, 0, 0, 0, 0, 0, 0, 0, 0, 0, 0, 0.232478632478632,

0, 0, 0, 0, 0, 0, 0, 0, 0, 0, 0, 0, 0, 0, 0, 0, 0, 0.857874134296899,

0, 0, 0, 0, 0, 0, 0, 0, 0, 0, 0, 0, 0, 0, 0, 0, 0, 0.0219285638496459,

0, 0, 0, 0, 0, 0, 0, 0, 0, 0, 0, 0, 0, 0, 0, 0, 0, 0.168131868131868,

0, 0, 0, 0, 0, 0, 0, 0, 0, 0, 0, 0, 0, 0, 0, 0, 0, 0.0826973577700322

), .Dim = c(17, 17))

**Example dataset EG2**

EG2 <- read.table(header=TRUE, as.is=TRUE, text="

study y ref trt contr design

1 -3.61988658 A B AB AB

2 0.00000000 B C BC BC

3 0.19342045 B C BC BC

4 2.79320801 B C BC BC

5 0.24512246 B C BC BC

6 0.03748309 B C BC BC

7 0.86020127 B D BD BD

8 0.14310084 B D BD BD

9 0.07598591 C D CD CD

10 -0.99039870 C D CD CD

11 -1.74085310 A B AB ABD

11 0.34830670 A D AD ABD

12 0.40546510 B C BC BCD

12 1.91692260 B D BD BCD

13 -0.32850410 B C BC BCD

13 1.07329450 B D BD BCD

")

S2 <- structure(c(0.9672619, 0, 0, 0, 0, 0, 0, 0, 0, 0, 0, 0, 0, 0,

0, 0, 0, 0.4, 0, 0, 0, 0, 0, 0, 0, 0, 0, 0, 0, 0, 0, 0, 0, 0,

0.24987648, 0, 0, 0, 0, 0, 0, 0, 0, 0, 0, 0, 0, 0, 0, 0, 0, 0.61904762,

0, 0, 0, 0, 0, 0, 0, 0, 0, 0, 0, 0, 0, 0, 0, 0, 0.27958937, 0,

0, 0, 0, 0, 0, 0, 0, 0, 0, 0, 0, 0, 0, 0, 0, 0.23845689, 0, 0,

0, 0, 0, 0, 0, 0, 0, 0, 0, 0, 0, 0, 0, 0, 0.04321419, 0, 0, 0,

0, 0, 0, 0, 0, 0, 0, 0, 0, 0, 0, 0, 0, 0.47692308, 0, 0, 0, 0,

0, 0, 0, 0, 0, 0, 0, 0, 0, 0, 0, 0, 0.18416468, 0, 0, 0, 0, 0,

0, 0, 0, 0, 0, 0, 0, 0, 0, 0, 0, 0.61978022, 0, 0, 0, 0, 0, 0,

0, 0, 0, 0, 0, 0, 0, 0, 0, 0, 0.12650164, 0.07397504, 0, 0, 0,

0, 0, 0, 0, 0, 0, 0, 0, 0, 0, 0, 0.07397504, 0.1583906, 0, 0,

0, 0, 0, 0, 0, 0, 0, 0, 0, 0, 0, 0, 0, 0, 0.389881, 0.2857143,

0, 0, 0, 0, 0, 0, 0, 0, 0, 0, 0, 0, 0, 0, 0.2857143, 0.5151261,

0, 0, 0, 0, 0, 0, 0, 0, 0, 0, 0, 0, 0, 0, 0, 0, 0.4361111, 0.2111111,

0, 0, 0, 0, 0, 0, 0, 0, 0, 0, 0, 0, 0, 0, 0.2111111, 0.5380342

), .Dim = c(16, 16))

**R code for fitting the models**

###### EXAMPLE 1 ######

### create contrast matrix

X <- contrmat(EG1$ref, EG1$trt)

### fit model assuming consistency (tau^2_omega=0)

modC <- rma.mv(y, S1, mods=X, intercept=FALSE, random = ~ contr | study, rho=1/2, data=EG1)

modC

### fit Jackson's model

modI <- rma.mv(y, S1, mods=X, intercept=FALSE, random = list(~ contr | study, ~ contr | design), rho=1/2, phi=1/2, data=EG1)

modI

### profile likelihood plots for tau^2_beta and tau^2_omega

par(mfrow=c(2,1))

profile(modI, tau2=1, xlim=c(0,.1))

abline(h=logLik(modI) - qchisq(.95, df=1)/2, lty="dotted")

profile(modI, gamma2=1, xlim=c(0,.8))

abline(h=logLik(modI) - qchisq(.95, df=1)/2, lty="dotted")

### profile likelihood CIs for tau^2_beta and tau^2_omega

confint(modI, tau2=1, digits=2)

confint(modI, gamma2=1, digits=2)

### LRT comparing modI and modC

anova(modI, modC)

###### EXAMPLE 2 ######

### create contrast matrix

X <- contrmat(EG2$ref, EG2$trt)

### fit model assuming consistency (tau^2_omega=0)

modC <- rma.mv(y, S2, mods=X, intercept=FALSE, random = ~ contr | study, rho=1/2, data=EG2)

modC

### fit Jackson's model

modI <- rma.mv(y, S2, mods=X, intercept=FALSE, random = list(~ contr | study, ~ contr | design), rho=1/2, phi=1/2, data=EG2)

modI

### profile likelihood plots for tau^2_beta and tau^2_omega

par(mfrow=c(2,1))

profile(modI, tau2=1, xlim=c(0,.1))

abline(h=logLik(modI) - qchisq(.95, df=1)/2, lty="dotted")

profile(modI, gamma2=1, xlim=c(0,.8))

abline(h=logLik(modI) - qchisq(.95, df=1)/2, lty="dotted")

### profile likelihood CIs for tau^2_beta and tau^2_omega

confint(modI, tau2=1, digits=2)

confint(modI, gamma2=1, digits=2)

### LRT comparing modI and modC

anova(modI, modC)

**Output**

###### EXAMPLE 1 ######

### model assuming consistency

Multivariate Meta-Analysis Model (k = 17; method: REML)

Variance Components:

outer factor: study (nlvls = 17)

inner factor: contr (nlvls = 9)

estim sqrt fixed

tau^2 0.0000 0.0000 no

rho 0.5000 yes

Test for Residual Heterogeneity:

QE(df = 10) = 2.2021, p-val = 0.9945

Test of Moderators (coefficient(s) 1,2,3,4,5,6,7):

QM(df = 7) = 7.3180, p-val = 0.3965

Model Results:

estimate se zval pval ci.lb ci.ub

B -0.2243 0.1072 -2.0926 0.0364 -0.4344 -0.0142 *

C -0.1667 0.2831 -0.5890 0.5558 -0.7216 0.3881

D -0.3274 0.3235 -1.0121 0.3115 -0.9615 0.3066

E -0.3152 0.2492 -1.2650 0.2059 -0.8036 0.1732

F -0.3520 0.2772 -1.2699 0.2041 -0.8952 0.1913

G -0.6489 0.8253 -0.7862 0.4317 -2.2665 0.9687

H -0.2758 0.4739 -0.5821 0.5605 -1.2046 0.6529

---

Signif. codes: 0 ‘***’ 0.001 ‘**’ 0.01 ‘*’ 0.05 ‘.’ 0.1 ‘ ’ 1

### Jackson's model

Multivariate Meta-Analysis Model (k = 17; method: REML)

Variance Components:

outer factor: study (nlvls = 17)

inner factor: contr (nlvls = 9)

estim sqrt fixed

tau^2 0.0000 0.0000 no

rho 0.5000 yes

outer factor: design (nlvls = 9)

inner factor: contr (nlvls = 9)

estim sqrt fixed

gamma^2 0.0000 0.0000 no

phi 0.5000 yes

Test for Residual Heterogeneity:

QE(df = 10) = 2.2021, p-val = 0.9945

Test of Moderators (coefficient(s) 1,2,3,4,5,6,7):

QM(df = 7) = 7.3180, p-val = 0.3965

Model Results:

estimate se zval pval ci.lb ci.ub

B -0.2243 0.1072 -2.0926 0.0364 -0.4344 -0.0142 *

C -0.1667 0.2831 -0.5890 0.5558 -0.7216 0.3881

D -0.3274 0.3235 -1.0121 0.3115 -0.9615 0.3066

E -0.3152 0.2492 -1.2650 0.2059 -0.8036 0.1732

F -0.3520 0.2772 -1.2699 0.2041 -0.8952 0.1913

G -0.6489 0.8253 -0.7862 0.4317 -2.2665 0.9687

H -0.2758 0.4739 -0.5821 0.5605 -1.2046 0.6529

---

Signif. codes: 0 ‘***’ 0.001 ‘**’ 0.01 ‘*’ 0.05 ‘.’ 0.1 ‘ ’ 1

### profile likelihood CIs for tau^2_beta and tau^2_omega

estimate ci.lb ci.ub

tau^2 0.00 0.00 0.07

tau 0.00 0.00 0.27

estimate ci.lb ci.ub

gamma^2 0.00 0.00 0.62

gamma 0.00 0.00 0.78

### LRT comparing modI and modC

df AIC BIC AICc logLik LRT pval QE

Full 9 19.1537 21.8769 199.1537 -0.5768 2.2021

Reduced 8 17.1537 19.5743 161.1537 -0.5768 0.0000 1.0000 2.2021

###### EXAMPLE 2 ######

### model assuming consistency

Multivariate Meta-Analysis Model (k = 16; method: REML)

Variance Components:

outer factor: study (nlvls = 13)

inner factor: contr (nlvls = 3)

estim sqrt fixed

tau^2 0.5482 0.7404 no

rho 0.5000 yes

Test for Residual Heterogeneity:

QE(df = 13) = 35.7508, p-val = 0.0006

Test of Moderators (coefficient(s) 1,2,3):

QM(df = 3) = 16.1343, p-val = 0.0011

Model Results:

estimate se zval pval ci.lb ci.ub

B -1.8847 0.6346 -2.9697 0.0030 -3.1286 -0.6408 **

C -1.3366 0.6927 -1.9294 0.0537 -2.6943 0.0212 .

D -0.7402 0.6633 -1.1159 0.2644 -2.0403 0.5599

---

Signif. codes: 0 ‘***’ 0.001 ‘**’ 0.01 ‘*’ 0.05 ‘.’ 0.1 ‘ ’ 1

### Jackson's model

Multivariate Meta-Analysis Model (k = 16; method: REML)

Variance Components:

outer factor: study (nlvls = 13)

inner factor: contr (nlvls = 3)

estim sqrt fixed

tau^2 0.1036 0.3219 no

rho 0.5000 yes

outer factor: design (nlvls = 6)

inner factor: contr (nlvls = 5)

estim sqrt fixed

gamma^2 0.5391 0.7342 no

phi 0.5000 yes

Test for Residual Heterogeneity:

QE(df = 13) = 35.7508, p-val = 0.0006

Test of Moderators (coefficient(s) 1,2,3):

QM(df = 3) = 13.6460, p-val = 0.0034

Model Results:

estimate se zval pval ci.lb ci.ub

B -1.9735 0.6762 -2.9183 0.0035 -3.2988 -0.6481 **

C -1.3957 0.8059 -1.7318 0.0833 -2.9753 0.1838 .

D -0.6572 0.7205 -0.9122 0.3617 -2.0694 0.7550

---

Signif. codes: 0 ‘***’ 0.001 ‘**’ 0.01 ‘*’ 0.05 ‘.’ 0.1 ‘ ’ 1

### profile likelihood CIs for tau^2_beta and tau^2_omega

estimate ci.lb ci.ub

tau^2 0.10 0.00 1.67

tau 0.32 0.00 1.29

estimate ci.lb ci.ub

gamma^2 0.54 0.00 3.96

gamma 0.73 0.00 1.99

### LRT comparing modI and modC

df AIC BIC AICc logLik LRT pval QE

Full 5 45.4920 48.3168 54.0635 -17.7460 35.7508

Reduced 4 44.5639 46.8237 49.5639 -18.2820 1.0719 0.3005 35.7508
